# Rapid determination of 103 common veterinary drug residues in milk and dairy products by ultra performance liquid chromatography tandem mass spectrometry

**DOI:** 10.3389/fnut.2022.879518

**Published:** 2022-07-22

**Authors:** Xiujuan Guo, Hao Tian, Fan Yang, Sufang Fan, Jingwen Zhang, Junmei Ma, Lianfeng Ai, Yan Zhang

**Affiliations:** ^1^Fourth Hospital of Hebei Medical University, Shijiazhuang, China; ^2^Technology Center of Shijiazhuang Customs District, Shijiazhuang, China; ^3^Hebei Food Inspection and Research Institute, Shijiazhuang, China; ^4^Hebei Key Laboratory of Forensic Medicine, College of Forensic Medicine, Hebei Medical University, Shijiazhuang, China

**Keywords:** rapid detection, veterinary drug residues, milk, dairy products, UPLC-MS/MS

## Abstract

A multi-residue method has been developed for the identification and quantification of 103 common veterinary drug residues in milk and dairy Products. This method was based on QuEChERS with dispersive solid-phase where C18 sorbent and anhydrous sodium sulfate were used to sample purification. After evaporation and reconstitution, the samples were analyzed by ultra-performance liquid chromatography-tandem mass spectrometry. The mean recovery results were all higher than 60% except ampicillin, pipemidic acid, enoxacin, and estriol, and the relative standard deviation was <20.0%. The limit of quantification ranged between 0.1 and 5 μg/kg for milk and between 0.5 and 25 μg/kg for milk powder. It was successfully used to detect residues of veterinary drug in real samples. This study proposes a simple and fast analytical method for monitoring multi-class veterinary drug residues to ensure food safety.

## Introduction

Milk and dairy products already enter thousands of households for their sweet and delicious taste, rich nutrition, easy digestion and absorption, and suitable for all ages in China. To protect milk consumers' health, it is essential to ensure that the products marketed are suitable. Because veterinary drugs are widely and sometimes incorrectly used in livestock breeding, residues of antibiotics, hormones, and anticoccidials, have been the main potential threats for the safety of milk and dairy products either as original compounds or metabolites. The presence of veterinary drug residues in food poses potential health risk to consumers as they could cause allergic reactions, present carcinogenic or teratogenic mechanisms, or induce antimicrobial resistance (1). Consequently, the highest residue of sulfonamides, quinolones, lincoamides, macrolides, β-lactam antibiotics, benzimidazoles, and triazines in the relevant laws and regulations formulated by many countries and regions such as China, European, and United States were at the μg/kg level (2–5). Chloramphenicol, nitroimidazole drugs and their metabolites, as well as some hormones, have potential animal carcinogenesis, teratogenicity, mutagenicity and genotoxicity, which have been listed as prohibited drugs or banned in dairy animals in many countries. Therefore, it is essential to monitor the drug residue in dairy products.

At present, the main methods for the determination of veterinary drug residues in animal-derived foods are high-performance liquid chromatography tandem mass spectrometry (HPLC-MS/MS) (6). The method for the determination of veterinary drug residue in a single category has been more mature. However, because most of the animal diseases are mixed infection, two or more different kinds of drugs are often used in clinical treatment. Therefore, the simultaneous detection of multiple residues of veterinary drugs is a new method studied in recent years (7–15). In sample preparation, most of the commonly used methods for the analysis of veterinary drug residue in foods were liquid-liquid extraction, solid phase extraction, but the operations were complicated and time-consuming. Quick, Easy, Cheap, Effective, Rugged, and Safe is a rapid pre-treatment method developed in recent years, which can be used to extract the target compound from the samples by a convenient, economical and rapid way (16–18). The main applications of quantitative multi-analyte methods in determination of veterinary drug residues in milk and other animal derived foods are presented in Table 1. In this work, 103 veterinary drugs, including sulfonamides, (fluoro)quinolones, nitroimidazoles and their metabolites, lincoamides, macrolides, β-lactams, benzimidazoles and their metabolites, exogenous estrogens, chloramphenicols, glucocorticoids, and triazines were simultaneously determined by QuEChERS combined with ultra-high performance liquid chromatography (UPLC) and tandem mass spectrometry (MS/MS). Compared with previous literatures, this work is more targeted and covers almost all possible veterinary drug contaminants in animal husbandry in China; isotope internal standard method is as much as possible used to qualify the analytes, then the result is more accurate; and the extraction and purification of target analytes with different chemical properties were realized by the simplest sample pretreatment method. This simple method, including sample extraction and data processing, allowed for high-throughput testing of milk samples to monitor veterinary drug residues.

**Table 1:** Comparison with other methods.

**Compounds**	**Matrix**	**Sample preparation**	**Detection identification**	**Quantitative method**	**Recoveries**	**Reference**
33 antibiotics: Sulfonamides (13), Tetracyclines (4), Macrolides (4), Quinolones (11), and Amphenicols (1)	Milk	Extraction and protein precipitation with ACN	LC-ESI-MS/MS (+) and (–)	Internal standard	–	(20)
18 veterinary drugs: Sulfonamides (4), Quinolones (2), Coccidiostats (7), Corticosteroids (3), Trimethoprim, and other contaminants (1)	Milk	Extraction with CAN–cleanup with SPE (Strata-X)	LC-ESI-MS/MS (+)	Internal standard and matrix external standard	65–119%	(21)
143 veterinary drugs and pharmaceuticals	Milk	Extraction with TCA 5% (w/v)–ACN (3:1, v/v), cleanup with HLB SPE	LC-ESI-Q/TOF (+)	External standard	>60%	(22)
	Fish	Extraction with 0.1% formic acid (v/v)/0.1% EDTA solution-MeOH-ACN, cleanup with hexane partitioning				
52 veterinary drugs, encompassing 12 classes: Aminocoumarins (1), amphenicols (2), anthelmintics (1), avermectins (4), imidazoles (9), lincosamides (2), macrolides (6), Non-steroidal anti inflammatory drugs (7), quinolones (2), β-lactams (8), sulfonamides (6), tetracyclines (2), and 2 unclassified compounds	Milk powders	Extraction with ACN, cleanup with Waters Oasis PRiME HLB	LC-ESI-MS/MS (+)	Internal standard	70–120%	(23)
84 veterinary drugs: Quinolones (14), Tetracyclines (4), Macrolides (7), β-Lactames (8), Sulfonamides (22), Trimethoprimethoprim, Tiamulin, Dapsone, Ormetoprim, Anthelmintics (21), and other contaminants (4)	Chicken muscle	Solid–liquid extraction with EDTA-succinate buffer and acetonitrile	LC-ESI-MS/MS (+)		29% (ofloxacin) to 98% (erythromycin)	(24)
155 veterinary drugs of 21 different classes	Animal source foods	Extraction with ACN–FA solution, cleanup with Waters Oasis PRiME HLB	LC-ESI-Q/Orbitrap (±)	External standard, add standard solution before purification	79.2–118.5%	(14)
103 veterinary drugs residuces: Nitroimidazoles (9), β-lactams (9), Licoamides (2), Macrolides (10), 20 (Fluoro)quinolones (20), Sulfonamides (22), Benzimidazoles (8), Exogenous estrogens (8), 3 Chloramphenicols (3), 8 Glucocorticoids (8), 4 triazines (4)	Milk and milk powder	Extraction with ACN, cleanup with improved QuEChERS	LC-ESI-MS/MS (+) and (–)	Internal standard and matrix external standard	>60% except ampicillin, pipemidic acid, enoxacin, and estriol	This work

## Materials and methods

### Instruments and reagents

WATERS ACQUITY UPLC, WATERS XEVO TQ-S tandem quadrupole mass spectrometry (Waters Corporation, USA); ACQUITY UPLC HSS T3 column (100 ^*^ 2.1 mm, 1.7 μm, Waters Corporation, USA), TDL-5-A centrifuge, water bath nitrogen blow-drying device, PT2100 type homogenizer, Vortex-Gene 2 scroll oscillator (United States Scientific Industries), 0.22. μm filter (Merck, Germany).

Among 103 common veterinary drug standards, including 22 sulfonamides, 20 (Fluoro)quinolones, 9 nitroimidazoles and metabolites, 9 β-lactams, 2 linconamides, 10 macrolides (species), 8 benzimidazoles and metabolites, 4 exogenous estrogens, 3 chloramphenicols, 8 glucocorticoids, 4 endogenous hormones, 4 triazines, and metabolites. Isotopic internal standards (sulfadimidine-D4, sulfathiazole-D6, sulfonamethoxazole-D6, sulfadimethoxypyrimidine-D4, sulfaquinoxane-D6, metronidazole-D4, hydroxymetronidazole-D2, hydroxymetronidazole-D3, metronidazole-D3, lonidazole-D3, hydroxyisopronidazole-D3, isoprolozole-D3norfloxacin-D5, ciprofloxacin-D8, enrofloxacin-D3, lonitrazole-D3, hydroxyisopronidazole-D3, isoprodazol-D3, norfloxacin-D5, ciprofloxacin-D8, enrofloxacin-D3 Chloramphenicol-D5, methyl prednisone-D4, 17-β-Estradiol-D4), the purity of standard compounds (seeing **Table 3** for details) ≥ 98%, all purchased by Dr Ehrenstorfer GmbH Company of Germany. N-propyl ethylenediamine adsorbents (PSA), octadecyl bonded silica gel adsorbents (C18) purchased from Waters Company of the United States. Methanol (HPLC grade), acetonitrile (HPLC grade), ethyl acetate (HPLC grade), formic acid (HPLC grade), anhydrous sodium sulfate (analytical regent), anhydrous magnesium sulfate (analytical regent), ultra-pure water were treated by Milli-Q water purification system (Millipore company). The constant volume solution was 0.1% formic acid water-acetonitrile solution (87 + 13, V/V) in positive mode and methanol aqueous solution (50 + 50, V/V) in negative mode. Preparation of standard storage solution and working liquid: the standard products were dissolved with methanol (β-lactam drugs in acetonitrile-water) and transferred to 100 mL brown capacity bottle for 10 mg, respectively. Dilute to 100 ml with methanol (or acetonitrile-water) and stored at −20°C. When used, the above standard stock solutions were mixed and diluted with acetonitrile into different concentrations of standard working solution.

### Sample preparation

The liquid milk was weighed 5.0 g, the precision was 0.01 g, and the milk powder sample was 1.0 g, mixed with 4.0 g water vortex and placed in a 50 mL centrifugal tube. After adding 25 μL 1.0 μg/mL internal standard solution (sulfonamides, quinolones, and nitroimidazole drugs using isotopic internal standard), 15.0 mL acetonitrile and 5 g anhydrous sodium sulfate were added accurately, the vortex was rotated rapidly for 2 min, and centrifuged at 4°C for 5 min for 5,000 r/min. The remaining part was added 10.00 mL acetonitrile, extracted at high speed with 10,000 r/min homogenizer for 2 min, centrifuged at 4°C for 5 min at 5,000 r/min, and combined with the liquid to be purified.

The sample was purified and removed at 6.00 mL in a 10 mL plug scale centrifugal tube. After adding 100 mg C18, 300 mg anhydrous sodium sulfate at one time, the vortex dispersed, and after centrifuging 5 min at 10,000 r/min, the 5.00 mL extract was accurately separated and blown to near dry at 45°C on the water bath nitrogen blowing dryer, and the concentrate was dissolved in 1.0 mL 0.1% formic acid water-acetonitrile. The solution filtered with 0.22 μm membrane, and used for UPLC-MS/MS analysis. According to the above steps, the blank extract of the sample was prepared.

### Instrument conditions

The UPLC conditions were column temperature of 40°C, injection volume of 5 μL; the positive ion scanning mode: flow rate of 0.3 mL/min. The mobile phase A is 0.1% formic acid solution, mobile phase B is acetonitrile, and the gradient elution conditions are shown in Table 2; the negative ion scanning mode: flow rate of 0.4 ml/min. The mobile phase A is pure water, mobile phase B is acetonitrile, and the gradient elution conditions are shown in Table 3.

**Table 2:** The conditions of gradient elution in ESI (+).

**Time**	**Flow rate**	**A**	**B**	**Gradient**
**(min)**	**(mL/min)**	**(%)**	**(%)**	**curve number**
0.0	0.3	95	5	6
4.5	0.3	85	15	6
5.0	0.3	85	15	6
6.0	0.3	80	20	6
6.5	0.3	75	25	8
8.5	0.3	50	50	6
10.0	0.3	5	95	6
10.5	0.3	0	100	6
10.6	0.3	95	5	6
12.6	0.3	95	5	6

**Table 3:** The conditions of gradient elution in ESI (–).

**Time**	**Flow rate**	**A**	**B**	**Gradient**
**(min)**	**(mL/min)**	**(%)**	**(%)**	**curve number**
0.00	0.4	80	20	–
0.50	0.4	85	15	6
2.00	0.4	85	15	6
4.00	0.4	80	20	6
9.50	0.4	75	25	8
11.00	0.4	50	50	6
11.10	0.4	5	95	6
11.60	0.4	95	5	1
13.00	0.4	95	5	1

The MS/MS conditions were electrospray ion source (ESI) including two methods. one was multi-reaction monitoring (MRM) mode scanning positive: electrospray voltage 3.0 kV, cone hole voltage 20 V, solvent removal gas temperature 450 °C, solvent removal gas flow rate 1,000 L/H, cone hole reverse blowing velocity 150 L/H, atomization gas pressure 7 Bar, ion source temperature 150°C. The other was MRM negative scanning mode: the electrospray voltage is 2.5 kV, cone hole voltage 30 V, the solvent removal gas temperature is 500°C, the solvent removal gas flow rate is 1,000 L/H, the cone hole reverse blowing velocity is 150 L/H, and the atomization gas pressure seven Bar, ion source temperature is 150°C. The parameters such as retention time and collision energy in positive and negative modes are shown in Table 4.

**Table 4:** Information and optimized mass spectrometry parameters of 103 standard veterinary drugs in ESI^+^ (1–80) and ESI^−^ (81–103).

**No**.	**Compounds**	**RT**	**Parent**	**Product**	**Collision energy**	**Scan time range**
		**(min)**	**(m/z)**	**(m/z)**	**(V)**	**(min)**
**9 Nitroimidazoles**						
1	Dimetridazole	3.64	142.1	81.0	22	0.10–4.30
				96.1^*^	14	
	Dimetridazole-IS	3.58	145.2	99.2	15	0.10–4.30
2	HMMNI	2.93	158.1	55.0	20	0.10–4.30
				140.1^*^	13	
	HMMNI-IS	2.92	161.2	143.1	11	0.10–4.30
3	Metronidazole	2.94	172.2	82.1	22	0.10–4.30
				128.0^*^	15	
	Metronidazole-IS	2.94	176.2	86.0	22	0.10–4.30
4	Metronidazole-OH	2.31	188.2	123.1^*^	12	0.10–4.30
				126.0	22	
	Metronidazole-OH-IS	2.37	190.2	128.0	19	0.10–4.30
5	Ronidazole	3.66	201.1	140.1^*^	12	0.10–4.30
	Ronidazole-IS	3.66	204.2	143.1	10	0.10–4.30
	Ipronuidazole	8.14	170.1	109.0	23	7.50–8.90
6				124.2^*^	17	
	Ipronidazole-IS	8.13	173.2	127.2	18	7.50–8.90
7	Ipronidazole-OH	6.43	186.2	122.0	18	6.10–8.00
				168.2^*^	14	
	Ipronidazole-OH-IS	6.4	189.2	125.2	18	6.10–8.00
8	Ornidazole	6.68	220.1	82.1	28	6.10–8.00
				128.0^*^	14	
9	Tinidazole	5.42	248.2	121.1^*^	16	5.20–6.50
				128.0	20	
**1 Cephalosporin**						
10	Ceftiofur	8.44	524.2	125.2	60	8.22–9.35
				241.1^*^	14	
**8 Penicillins**						
11	Penicillin V	9.34	351.2	114.1	32	9.10–11.20
				160.0^*^	12	
12	Oxacillin	9.58	402.2	160.1^*^	12	9.10–11.20
				243.2	12	
13	Nafcillin	9.86	415.2	171.1	38	9.10–11.20
				199.1^*^	14	
14	Cloxacillin	9.76	436.0	159.8^*^	20	9.10–11.20
				277.0	16	
15	Dicioxacillin	10.02	470.2	159.8^*^	16	9.10–11.20
				311.1	13	
16	Penicillin G	5.85	335.2	91.0^*^	40	5.20–6.50
				128.0	26	
17	Ampicillin	4.92	350.2	106.1^*^	16	4.20–5.50
				160.1	10	
18	Piperacillin	8.78	518.3	143.1^*^	19	8.35–9.70
				160.1	10	
**2 Licoamides**						
19	Clindamycin	8.02	425.1	125.9^*^	27	7.50–8.90
				377.1	17	
20	Lincomycin	4.38	407.1	125.9^*^	29	4.20–5.50
				359.2	17	
**10 Macrolides**						
21	Tilmicosin	8.11	869.6	174.0^*^	42	7.50–8.90
				696.6	41	
22	Josamycin	9.45	828.8	109.1	35	9.10–11.20
				174.2^*^	32	
23	Tamulin	9.11	494.4	119.0	32	8.35–9.70
				192.1^*^	20	
24	Oleandomycin	9.14	688.7	158.2^*^	27	8.35–9.70
				544.6	16	
25	Erythromycin	8.69	734.4	158.0^*^	30	8.35–9.70
				576.5	17	
26	Stereomycin	9.1	772.4	108.8^*^	37	8.35–9.70
				215.0	28	
27	Roxithromycin	9.29	837.6	158.2^*^	33	8.35–9.70
				679.5	22	
28	Tylosin	8.84	916.5	100.8	43	8.35–9.70
				174.0^*^	38	
29	Azithromycub	7.81	749.5	158.0^*^	40	7.05–8.20
				591.5	29	
30	Spriamycin	7.76	843.5	141.9	33	7.05–8.20
				174.0^*^	35	
**20 (Fluoro)quinolones**						
31	Cinoxacin	8.09	263.0	189.0	26	7.50–8.90
				245.0^*^	15	
32	Lomefloxaxin	6.6	352.1	208.2	22	6.10–7.60
				265.0^*^	16	
33	Danofloxacin	6.7	358.2	314.1^*^	17	6.10–7.60
				340.1	22	
34	Enrofloxacin	6.95	360.1	245.0	26	6.10–7.60
				316.2^*^	18	
	Enrofloxacin-IS	6.93	364.9	321.0	20	6.10–7.60
35	Orbifloxacin	7.1	396.3	295.2^*^	24	6.10–7.60
				352.6	17	
36	Nalidixic acid	9.25	233.0	186.9	24	9.10–11.20
				215.0	14	
37	Flumequine	9.39	262.1	202.0	32	9.10–11.20
				244.0^*^	21	
38	Norfloxaxin	5.81	320.0	233.0	22	5.20–6.50
				276.2^*^	15	
	Norfloxaxin-IS	5.81	324.9	281.0	16	5.20–6.50
39	Enoxacin	5.52	321.1	232.0	36	5.20–6.50
				303.1^*^	15	
40	Ciprofloxacin	6.13	332.0	245.0	21	5.20–6.50
				288.2^*^	16	
	Ciprofloxacin-IS	6.13	339.9	296.0	18	5.20–6.50
41	Pefloxacin	6.03	334.1	290.1^*^	17	5.20–6.50
				316.1	19	
42	Ofloxacin	5.89	362.1	261.1	25	5.20–6.50
				318.1^*^	19	
43	Marbofloxacin	5.43	363.1	71.8	20	5.20–6.50
				320.0^*^	15	
44	Flerofloxacin	5.78	370.1	269.1	25	5.20–6.50
				326.1^*^	19	
45	Pipemidic acid	4.53	304.0	217.0^*^	21	4.20–5.50
				286.1	18	
46	Oxolinic acid	8.46	262.0	216.0	30	8.20–9.35
				244.0^*^	19	
47	Gatifloxacin	7.33	376.1	289.1	28	7.05–8.20
				332.1^*^	19	
48	Sarafloxacin	7.47	386.1	299.1	26	7.05–8.20
				342.2^*^	16	
49	Sparfloxaxin	7.59	393.1	292.1	24	7.05–8.20
				349.2^*^	20	
50	Difloxaxin	7.54	400.1	299.1	26	7.05–8.20
				356.2^*^	18	
**22 Sulfonamides**						
51	Sulphanilamide	1.71	173.8	65.6	30	0.10–4.30
				92.8^*^	22	
52	Sulfaguanidine	1.42	214.8	91.8^*^	24	0.10–4.30
				155.8	14	
53	Sulfacetamid	3.23	215.0	107.8^*^	18	0.10–4.30
				155.8	8	
54	Sulfadiazine	3.85	251.0	91.8	27	0.10–4.30
				155.8^*^	15	
	Sulfadiazine-IS	3.85	156.8	161.8	14	0.10–4.30
55	Sulfamethoxazole	7.96	254.0	91.8	26	7.70–8.50
				155.8^*^	16	
	Sulfamethoxazole-IS	7.96	259.8	161.8	14	7.70–8.50
56	Sulfamoxole	8.21	268.0	91.8	23	7.70–8.50
				155.8^*^	15	
57	Sulfadoxine	7.95	311.0	91.8	32	7.70–8.50
				155.8^*^	15	
58	Sulfamethizole	6.23	271.0	91.8	30	5.95–7.15
				155.8^*^	15	
59	Sulfadimidine	6.25	279.0	91.8^*^	30	5.95–7.15
				124.0	20	
60	Sulfameter	6.43	281.0	125.8	20	5.95–7.15
				155.8^*^	16	
61	Sulfamethoxypyridazine	6.55	281.0	125.8	20	5.95–7.15
				155.8^*^	16	
62	Sulfisoxazole	5.79	268.0	91.8	25	5.05–6.35
				155.8^*^	13	
63	Sulfapyridine	4.59	250.0	107.8	22	4.0–5.35
				155.8^*^	14	
64	Sulfathiazole	4.35	256.0	91.8	25	4.0–5.35
				155.8^*^	15	
	Sulfathiazole-IS	4.34	261.8	161.8	14	4.0–5.35
65	Sulfamerazine	5.06	265.0	91.8^*^	28	4.0–5.35
				155.8	15	
66	Trimethoprim	5.22	291.0	122.8^*^	27	4.0–5.35
				229.8	25	
67	Sulfabenzamide	8.54	277.0	107.8	22	8.2–9.35
				155.8^*^	15	
68	Sulfaquinoxaline	8.73	301.1	91.8	27	8.2–9.35
				155.8^*^	15	
	Sulfaquinoxaline-IS	8.73	306.9	161.8	16	8.2–9.35
69	Sulfadimethoxine	8.72	311.1	91.8	32	8.2–9.35
				155.8^*^	20	
	Sulfadimethoxine-IS	8.66	316.9	161.9	22	8.2–9.35
70	Sulfaphenazole	8.78	315.0	155.8	19	8.2–9.35
				159.8^*^	20	
71	Sulfamonomethoxine	7.37	281.0	125.8	20	7.0–8.0
				155.8^*^	16	
72	Sulfachloropyridazine	7.6	285.1	91.8	28	7.0–8.0
				155.8^*^	15	
**8 Benzimidazoles**						
73	Albendazole sulfone	8.3	298.0	159.0^*^	25	7.5–8.9
				266.0	18	
74	Oxfendazole	8.21	316.2	159.1^*^	32	7.5–8.9
				191.2	20	
75	Albendazole S-oxide	7.32	282.3	159.2	32	6.1–8.00
				208.1^*^	20	
76	Fenbendazole	9.87	300.2	159.2	20	9.10–11.2
				268.8^*^	20	
77	Flubendazole	9.45	314.1	122.9	33	9.10–11.2
				282.1^*^	22	
78	Fenbendazole sulfone	9.07	332.2	159.1	42	8.5–9.7
				300.2^*^	20	
79	Mebendazole	8.99	296.3	105.1	34	8.5–9.7
				264.2^*^	20	
80	Albendazole	9.23	266.2	191.3	32	8.5–9.7
				234.2^*^	20	
**4 Exogenous estrogens**						
81	Zeranol	8.25	321.3	161.0	−32	8.05–10.50
				277.3^*^	−24	
82	Hexestrol	9.91	269.2	119.0^*^	−40	8.05–10.50
				134.1	−14	
83	Diethyatibestrol	9.02	267.2	237.1	−30	8.05–10.50
				251.2^*^	−26	
	Diethyatibestrol-D8	9	275.2	245.1^*^	−28	8.05–10.50
84	Dienestrol	9.08	265.2	93.0^*^	−24	8.05–10.50
				235.2	−40	
**3 Chloramphenicols**						
85	Chloramphenicol	3.47	321.0	152.1^*^	−18	0.10–4.00
				257.1	−12	
	Chloramphenicol-D5	3.51	326.1	157.1	−18	0.10–4.00
86	Thiamphenicol	1.56	354.0	184.9^*^	−24	0.10–4.00
				227.0	−14	
87	Florfeniol	2.91	356.0	184.9	−20	0.10–4.00
				336.0^*^	−8	
**8 Glucocorticoids**						
88	Hydrocortisone	4.82	407.1	297.1	−28	4.10–7.60
				331.1^*^	−16	
89	Prednisolone	4.64	403.1	295.0	−28	4.10–7.60
				329.1^*^	−11	
90	Prednisone	4.62	430.1	299.1	−18	4.10–7.60
				327.1^*^	−13	
91	Beclomethasone	7.17	453.1	377.1^*^	−13	4.10–7.60
				407.1	−11	
92	Betamethasone	6.56	437.1	307.0	−30	4.10–7.60
				361.1^*^	−18	
93	Dexamethasone	6.73	437.1	307.0	−30	4.10–7.60
				361.1^*^	−18	
94	Fludrocortisone	4.9	425.5	295.1	−33	4.10–7.60
				349.1^*^	−20	
95	Methylprednisolone	6.35	419.1	309.0	−32	4.10–7.60
				343.1^*^	−18	
	Methylprednisolone-D4	6.34	422.4	346.3	−14	4.10–7.60
**4 Endogenous estrogens**						
96	Ethinylestradiol	8.68	295.2	145.1	−40	7.80–9.00
				267.2	−25	
97	Estriol	3.54	287.2	145.1	−42	7.80–9.00
				171.1	−36	
98	17-α-estradiol	8.51	271.2	145.1	−40	7.80–9.00
				183.1	−38	
99	17-β-estradiol	8.24	271.2	145.1	−40	7.80–9.00
				183.1	−38	
	17-β-estradiol-D4	8.19	275.2	187.2	−38	7.80–9.00
**4 triazines**						
100	Toltrazuril	9.59	424.3	424.3^*^	−4	8.20–10.0
	Toltrazuril-D3	9.58	427.0	427.0^*^	−4	8.20–10.0
101	Toltrazuril sulfoxide	8.55	440.2	371.2^*^	−17	8.20–10.0
				383.2	−13	
102	Toltrazuril sulfone	9.19	456.0	456.0^*^	−4	8.20–10.0
103	Diclazuril	9.41	405.0	334.0^*^	−19	8.20–10.0

* *is a quantitative ion*.

## Results

### Selection of extraction solvents

For the simultaneous determination of veterinary drug residues in milk and milk powder, the extraction method is the key. According to the different chemical properties of each compound, the recovery rates of each target substance were investigated when methanol, acetonitrile, ethyl acetate, and methanol-acetonitrile (1:1) were used as extractants, respectively. It was found that when extracted with ethyl acetate and methanol solution, the fat was too much, the effect of removing impurities was not good, and the recovery rate of penicillin drugs was not easy to be concentrated by nitrogen blowing, and the recovery rate of penicillin drugs was not easy to be controlled when extracted with methanol-acetonitrile (1:1), only nitroimidazole drugs and some sulfonamides and (fluoro)quinolones were recovered in the positive mode, and the protein precipitation was not complete. When acetonitrile is a polar solvent, it can avoid extracting too much fat from the tissue and has a good protein deposition effect at the same time. Therefore, it was preliminarily determined that acetonitrile was used as extraction solvent. Because sulfonamides, nitroimidazoles, and quinolones are amphoteric substances, they are more easily extracted in acidic extraction solvents. Macrolides, lincosamides drugs and benzimidazole drugs belong to weak basic substances, and the pKa values of all kinds of drugs are quite different. In this study, the extraction efficiency of pure acetonitrile, 1% formic acid and 1% acetic acid were compared. The effect of 1% formic acid and 1% acetic acid on the extraction efficiency of all kinds of drugs was not consistent. The extraction efficiency of (fluoro)quinolones with acidic acetonitrile was better, but the extraction rate of erythromycin decreased under acidic acetonitrile, which may be due to the hydrolysis of glycoside bonds. However, considering that the same pretreatment method should be shared with the negative model, acidity has a great influence on the substance of the negative mode. Considering the factors affecting the extraction efficiency and protein removal of all kinds of drugs, especially the effect on the stability of drugs under acidic and alkaline conditions, acetonitrile was used to extract all kinds of drugs without adding acid. Supplementary Figure 1 showed the results of typical compounds with different chemical properties using different extractions. When the sample is extracted, proper amount of anhydrous sodium sulfate can effectively prevent the moisture and water-soluble impurities in the sample from entering the extract solution. Sodium sulfate also has salting out effect, which is helpful to improve the extraction efficiency. Then, in the extraction add 5 g anhydrous sodium sulfate and extract twice with acetonitrile.

### Selection of purification conditions

Two purification methods, liquid-liquid partition extraction and solid phase extraction, were compared. Waters HLB column, MCX column and MAX column were used to purify the compounds, but because there were too many kinds of veterinary drugs, the properties of the compounds varied greatly and were not easy to be controlled (see Supplementary Figure 2). For example, sulfonamides were easy to be lost after solid phase extraction column, and the recovery rate was on the low side. The results of SPE Therefore, in this experiment, the purification method of SPE was abandoned, and the dispersed solid phase extraction (QuEChERS) with less time consuming and low analysis cost was selected. The general rapid pretreatment method of 103 compounds should not only consider the recoveries, but also effectively purify the matrix and reduce the influence of impurities. However, the existing commercial QuEChERS adsorbents or purification tubes are not suitable for the pretreatment of various drug residue with different physical and chemical properties. Commonly used adsorbents include C18, primary secondary amine (PSA), Flori silica, and graphitized carbon black (GCB). When adsorbing impurities, the adsorbents may also adsorb the target compounds, which may affect the recovery rate of the target compounds. Therefore, when selecting the adsorbents, we should try our best to select the adsorbents which have little influence on the target compounds. As a strong polar absorbent, Flori silica cannot remove lipid and carbohydrate impurities; GCB is usually used to remove pigment components from plant extract, because it can adsorb strong drugs with benzene ring functional groups, which has a great impact on the recovery of most penicillin, quinolones, sulfonamides and benzimidazoles; PSA can effectively remove fatty acids and sterols from the matrix, but there are fat residue in the purified extract, and PSA can adsorb acidic drugs as alkaline adsorption fillers. C18 can effectively remove lipophilic impurities such as lipids and carbohydrates, but excessive C18 can also adsorb lipophilic drugs. Therefore, combined with the above factors, the purification effect of C18 and anhydrous sodium sulfate was investigated. The C18 of 20, 50, 100, 200, 500 mg was used for purification, and it was found that when the amount of C18 powder was added, the solution was mixed into a yellowish clear solution. Although the amount of C18 powder was too much, the purification effect was good, but some of the target substances were adsorbed (see Supplementary Figure 3). Therefore, a good balance between purification effect and recovery can be obtained by using mixed adsorbents of 100 mg C18 and 300 mg anhydrous sodium sulfate per gram of sample. The extract optimized by QuEChERS was yellowish and transparent, and the overall recovery was 31.1–120.7%.

### HPLC conditions

**Figure 1:**
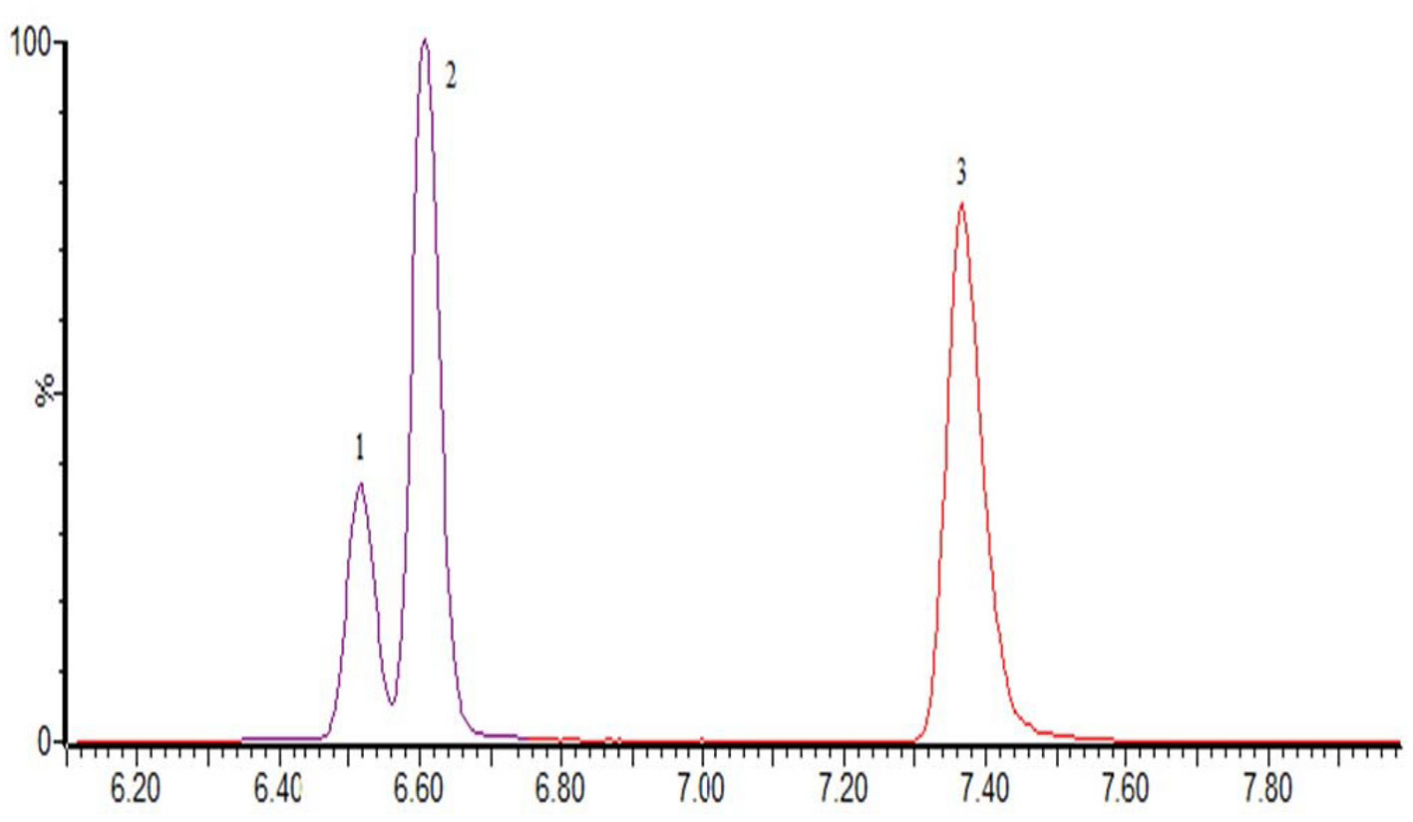
The MRM of sulfameter (1), sulfamethoxypyridazine (2), and sulfamonomethoxine (3) in positive mode.

**Figure 2:**
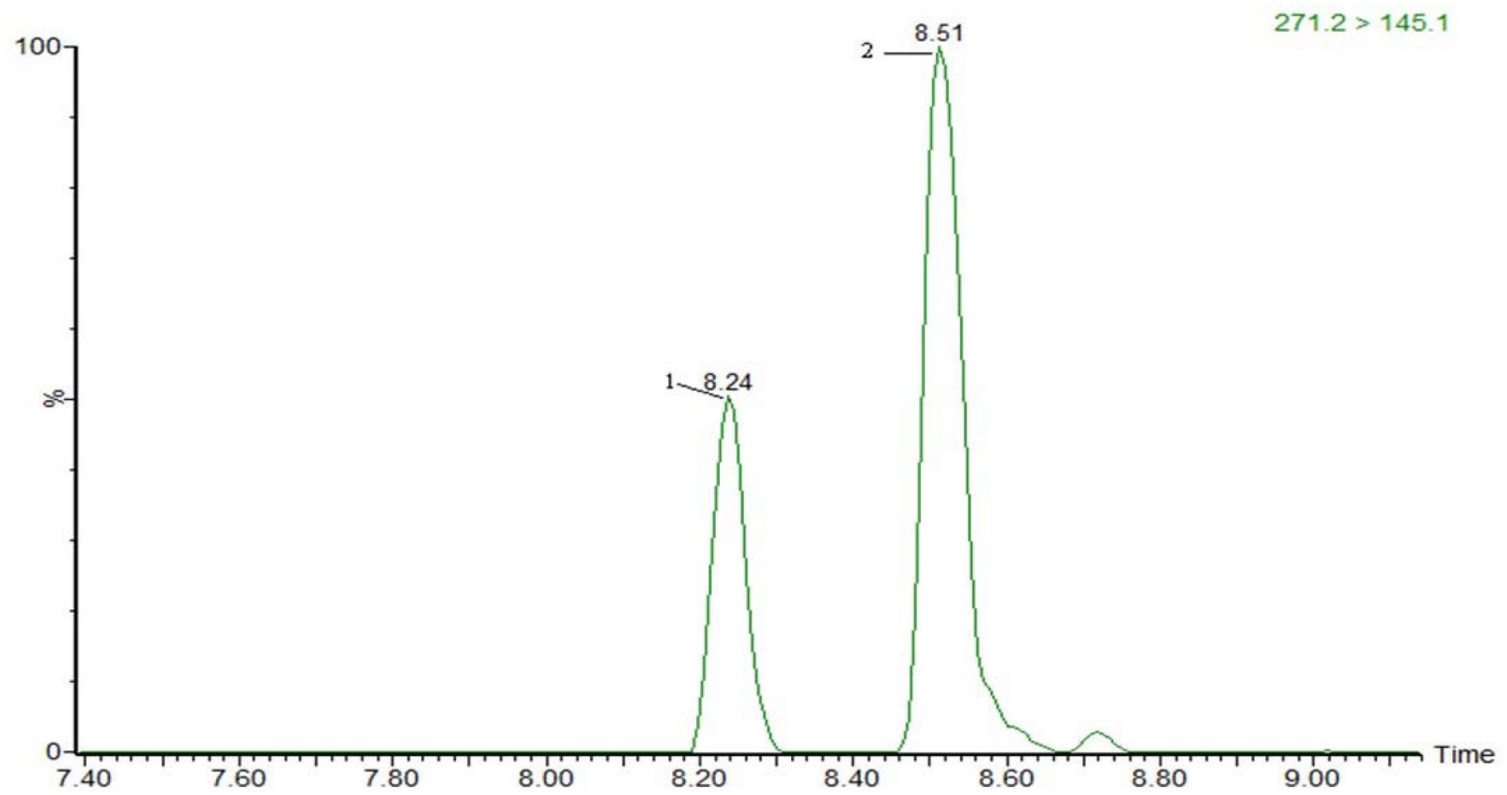
The MRM of 17-α-estradiol (2) and 17-β-estradiol (1) in negative mode.

The optimization of the separation condition mainly considers the influence of the matrix, the separation effect, and the response strength. The ACQUITY UPLC BEH C18 column (100 ^*^ 2.1 mm, 1.7 μm) was compared when the column was selected. and HSS T3 (100 ^*^ 2.1 mm, 1.8 μm). In the positive mode, the separation condition can be met by changing the elution condition of the mobile phase by continuously changing the elution condition of the mobile phase. However, because of the large polarity of some substances in the penicillins, the stability and the linearity of this kind of substance have obvious advantages when using T3 column. Therefore, HSS T3 is selected as analytical column, and the co-isomer of the positive mode scanning is taken as an example for the separation of sulfameter, sulfamethoxypyridazine, and sulfamonomethoxine. The separation chromatograms were shown in Figure 1; the negative pattern can be separated from the isomer by the HSS T3 column, and the separation degree can be maintained. Take estradiol as an example, seeing Figure 2.

In the mobile phase selection, methanol and acetonitrile were selected as the strong eluting mobile phase. The experimental results show that when methanol is used as mobile phase, the ionization of some drugs is inhibited to varying degrees, the peak shape widens, the abundance decreases obviously, and the sensitivity decreases, while the ionization efficiency of acetonitrile is obviously better than that of methanol, so the mobile phase is strongly eluted with acetonitrile. In positive mode, 0.1% formic acid solution, aqueous solution and 5 mmol/L amine acetate (containing 0.1% acetic acid) were selected as aqueous phase (A), acetonitrile as organic phase (B). The mobile phase selection of UPLC was carried out. The results showed that the peak shape of sulfonamides and quinolones was wide when aqueous solution was used as phase A, penicillin was unstable and some substances were poor when 5 mmol/L acetic acid (containing 0.1% acetic acid) was used as phase A when 0.1% formic acid solution was used as phase A, most of the substances such as sulfonamides quinolones were in ion state, the peak shape was sharp symmetry, and the signal response of mass spectrometry was enhanced. In negative mode, water, 0.1% ammonia aqueous solution and 0.5 mmol/mL ammonium acetate solution were selected as aqueous phase (A), acetonitrile as organic phase (B) to select UPLC mobile phase. Because some compounds in the negative model, especially the above factors, were selected as the mobile phase of the method, 0.1% formic acid water and acetonitrile were selected as the mobile phase and eluted under gradient conditions.

### Mass spectrometry conditions

**Figure 3:**
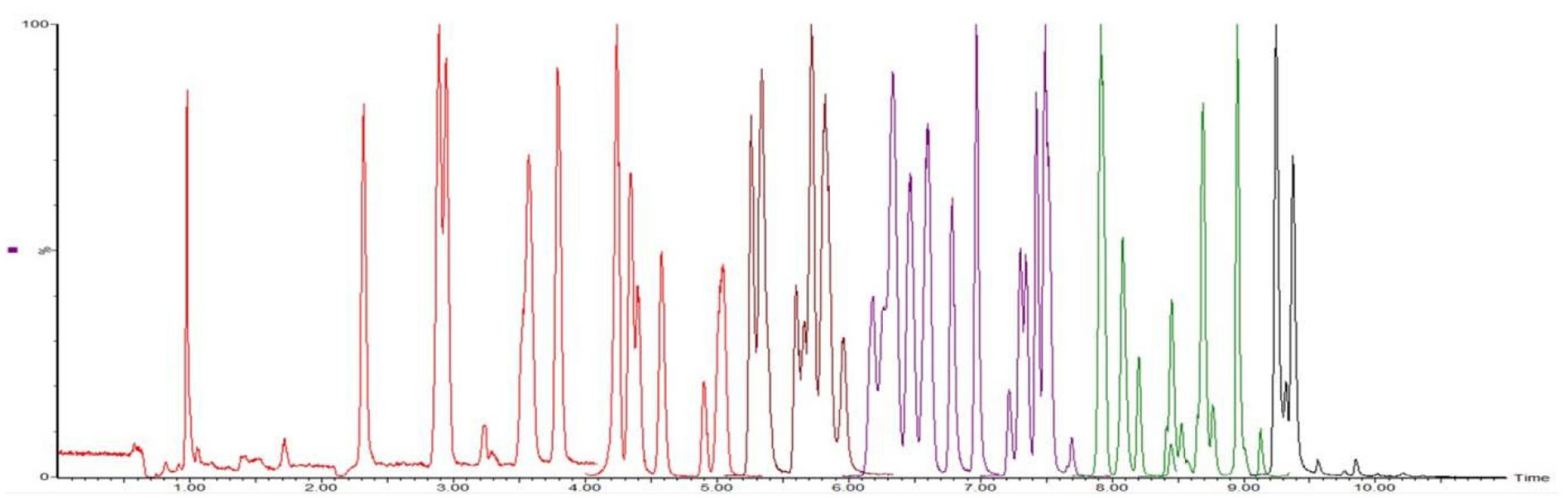
The TIC of 80 analytes mixed drugs standard solution in milk (ES+).

**Figure 4:**
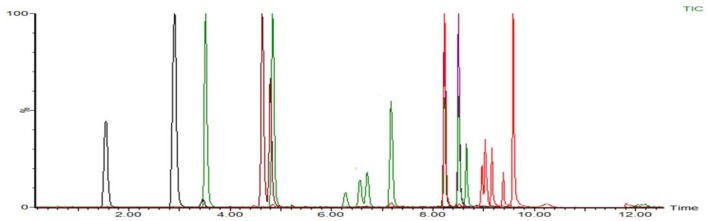
The TIC of 23 analytes mixed drugs standard solution in milk (ES–).

According to the chemical structure of the analytes to be tested, these 12 kinds of analytes are suitable for ionization in the positive ion mode of ESI source. One hundred three kinds of standards were diluted with acetonitrile into a mixed solution of 0.1 μg/mL and injected into peristaltic pump. The responses of 80 analytes in positive ion scanning modes were higher than in the negative ion scanning modes, and for the other 23 analytes were the reverse. The all parent ions were all molecular ion of [M+H] + or [M–H]–, except glucocorticoids, for which the ions of [M+HCOOH-H]- have the best responses. The daughter ions were analyzed by full scan, and two characteristic daughter ions were selected, in which the ion pairs with high signal to noise ratio, good peak shape, and small interference were used as quantitative ions. The total ion chromatograms (TIC) current of 103 drugs in added milk are illustrated in Figures 3, 4 under the conditions of positive and negative mode, respectively. The MRM chromatograms of every compound were showed in Supplementary Figure 4.

### Method validation

#### Linear range, linear equation, and detection limit

The six levels of the series of mixed matrix working solutions were determined under the selected chromatographic separation conditions and mass spectrometry parameters. the ratio of the peak area of the target to the peak area of the internal standard substance is the vertical coordinate (Y), and the mass concentration (ng/mL) of the target substance is the abscissa (X) as the quantitative working curve; The of the matrix was obtained, the linear correlation coefficients linear regression equation ranged from 0.9902 to 0.9998, indicating that the compound has a good linear relationship within the corresponding concentration range. The detection limit of the compound (LOD) and the limit of quantitation (LOQ) were determined according to the 3 and 10 signal-to-noise (S/N) ratio. The LODs of 103 drugs in the milk were 0.1–10 μg /kg, and the LOQs were 0.5–25 μg /kg; the LODs in the milk powder matrix are 0.5–25 μg /kg, and the LOQs are 0.5–50 μg/kg. The results are shown in Table 5.

**Table 5:** Calibration curve, linear ranges, correlation coefficients, LODs, and LOQs.

**No**.	**Compound**	**Matrix**	**ng/mL**	**Calibration curve**	**R**	**LOD** **μg/kg**	**LOQ** **μg/kg**
**9 Nitroimidazoles**							
1	Dimetridazole	Milk	0.5–100	Y = 0.639X + 0.122	0.9959	0.25	0.5
		Milk powder	0.5–100	Y = 0.720X + 0.043	0.9995	1.25	2.5
2	HMMNI	Milk	1.0–200	Y = 0.696X + 0.301	0.9967	0.5	1
		Milk powder	1.0–200	Y = 0.686X + 0.088	0.9979	2.5	5
3	Metronidazole	Milk	0.5–100	Y = 1.788X + 0.361	0.9957	0.25	0.5
		Milk powder	0.5–100	Y = 1.812X + 0.040	0.9992	1.25	2.5
4	Metronidazole-OH	Milk	1.0–200	Y = 1.105X + 0.540	0.9968	0.5	1
		Milk powder	1.0–200	Y = 1.419X + 0.311	0.9964	2.5	5
5	Ronidazole	Milk	0.5–100	Y = 0.767X + 0.158	0.9959	0.25	0.5
		Milk powder	0.5–100	Y = 0.843X + 0.013	0.9993	1.25	2.5
6	Ipronidazole	Milk	0.5–100	Y = 0.819X + 0.146	0.9982	0.25	0.5
		Milk powder	0.5–100	Y = 0.793X + 0.156	0.9956	1.25	2.5
7	Ipronidazole-OH	Milk	0.5–100	Y = 2.152X + 0.359	0.9976	0.25	0.5
		Milk powder	0.5–100	Y = 2.279X + 0.527	0.9956	1.25	2.5
8	Ornidazole^*^	Milk	0.5–100	Y = 92,308X + 6,667	0.9962	0.25	0.5
		Milk powder	0.5–100	Y = 80,354X + 11,838	0.9969	1.25	2.5
9	Tinidazole^*^	Milk	0.5–100	Y = 86,108X + 6,759	0.9956	0.25	0.5
		Milk powder	0.5–100	Y = 80,354X + 11,838	0.9969	1.25	2.5
**9 β-lactams**							
10	Ceftiofur^*^	Milk	10–100	Y = 213.5X + 42.82	0.9990	2.5	10
		Milk powder	10–100	Y = 423.1X−6.541	0.9982	12.5	50
11	Penicillin V^*^	Milk	10–100	Y = 272.1X + 467.5	0.9943	2.5	10
		Milk powder	10–100	Y = 4,806X + 4,720	0.9943	12.5	50
12	Oxacillin^*^	Milk	10–100	Y = 1,381X−191.4	0.9976	2.5	10
		Milk powder	10–100	Y = 2,096X + 3,707	0.9936	12.5	50
13	Nafcillin^*^	Milk	10–100	Y = 1749X−58.15	0.9964	2.5	10
		Milk powder	10–100	Y = 3,077X + 6,349	0.9933	12.5	50
14	Cloxacillin^*^	Milk	10–100	Y = 390.7X−24.30	0.9974	2.5	10
		Milk powder	10–100	Y = 692.2X + 1,425	0.9911	12.5	50
15	Dicioxacillin^*^	Milk	10–100	Y = 168.9X−35.42	0.9941	2.5	10
		Milk powder	10–100	Y = 413.5X + 790.1	0.9978	12.5	50
16	Penicillin G^*^	Milk	10–100	Y = 4806X + 4,720	0.9972	2.5	10
		Milk powder	10–100	Y = 2,530X−686.9	0.9926	12.5	50
17	Ampicillin^*^	Milk	10–100	Y = 19,085X + 2,353	0.9963	2.5	10
		Milk powder	10–100	Y = 16,238X−2,682	0.9912	12.5	50
18	Piperacillin^*^	Milk	10–100	Y = 2,925X + 2.353	0.9993	2.5	10
		Milk powder	10–100	Y = 2,456X−221.0	0.9966	12.5	50
**2 Lincosamides**							
19	Clindamycin^*^	Milk	0.25–50	Y = 76,561X + 15,921	0.9972	0.25	0.25
		Milk powder	0.25–50	Y = 102,009X + 68.8	0.9929	1.25	1.25
20	Lincomycin^*^	Milk	0.25–50	Y = 97,200X + 17,976	0.9963	0.25	0.25
		Milk powder	0.25–50	Y = 137,158X−2,470	0.9972	1.25	1.25
**10 Metabolites**							
21	Tilmicosin^*^	Milk	0.5–100	Y = 1,446X + 202.1	0.9965	0.25	0.5
		Milk powder	0.5–100	Y = 1,699X−93.10	0.9979	1.25	2.5
22	Josamycin^*^	Milk	0.5–100	Y = 18,323X + 2,412	0.9951	0.25	0.5
		Milk powder	0.5–100	Y = 19,955X + 679.9	0.9992	1.25	2.5
23	Tamulin^*^	Milk	0.25–30	Y = 1,446X + 202.1	0.9944	0.25	0.25
		Milk powder	0.25–30	Y = 19,001X−12,352	0.9978	1.25	1.25
24	Oleandomycin^*^	Milk	0.5–100	Y = 16,339X + 28,48	0.9965	0.25	0.5
		Milk powder	0.5–100	Y = 17,984X + 1,238	0.9947	1.25	2.5
25	Erythromycin^*^	Milk	2.0–100	Y = 1,646X + 207.9	0.9915	0.5	2
		Milk powder	2.0–100	Y = 3,349X + 1,521	0.9918	2.5	10
26	Stereomycin^*^	Milk	0.5–100	Y = 6,124X + 1,213	0.9961	0.25	0.5
		Milk powder	0.5–100	Y = 8,190X + 5.480	0.9946	1.25	2.5
27	Roxithromycin^*^	Milk	0.5–100	Y = 15,726X + 2,810	0.9958	0.25	0.5
		Milk powder	0.5–100	Y = 15,638X + 283.1	0.9955	1.25	2.5
28	Tylosin^*^	Milk	0.5–100	Y = 9,936X + 1,637	0.9942	0.25	0.5
		Milk powder	0.5–100	Y = 12,202X + 71.16	0.9967	1.25	2.5
29	Azithromycub^*^	Milk	0.5–100	Y = 2,486X + 0.9926	0.9926	0.25	0.5
		Milk powder	0.5–100	Y = 2,985X−105.8	0.9978	1.25	2.5
30	Spriamycin^*^	Milk	0.5–100	Y = 1,208X + 144.6	0.9951	0.25	0.5
		Milk powder	0.5–100	Y = 1,347X−44.71	0.9971	1.25	2.5
**20 (Fluoro)quinolones**							
31	Cinoxacin	Milk	1.0–100	Y = 2.336X + 0.426	0.9968	0.25	1
		Milk powder	1.0–100	Y = 2.559X + 0.479	0.9954	1.25	5
32	Lomefloxaxin	Milk	1.0–100	Y = 1.231X + 0.154	0.9976	0.25	1
		Milk powder	1.0–100	Y = 1.298X−0.131	0.9943	1.25	5
33	Danofloxacin	Milk	1.0–100	Y = 0.415X + 0.072	0.9975	0.25	1
		Milk powder	1.0–100	Y = 0.482X−0.059	0.9949	1.25	5
34	Enrofloxacin	Milk	1.0–100	Y = 2.404X + 0.432	0.9959	0.25	1
		Milk powder	1.0–100	Y = 2.319X−0.024	0.9955	1.25	5
35	Orbifloxacin	Milk	1.0–100	Y = 2.563X + 0.476	0.9954	0.25	1
		Milk powder	1.0–100	Y = 2.498X + 0.083	0.9956	1.25	5
36	Nalidixic acid	Milk	1.0–100	Y = 7.482X + 1.548	0.9986	0.25	1
		Milk powder	1.0–100	Y = 6.737X + 0.745	0.9958	1.25	5
37	Flumequine	Milk	0.5–50	Y = 5.187X + 0.998	0.9976	0.25	0.5
		Milk powder	0.5–50	Y = 6.015X + 1.438	0.9947	1.25	5
38	Norfloxaxin	Milk	1.0–100	Y = 2.337X + 0.393	0.9957	0.25	1
		Milk powder	1.0–100	Y = 2.139X−0.281	0.9984	1.25	5
39	Enoxacin	Milk	1.0–100	Y = 0.667X + 0.064	0.9986	0.25	1
		Milk powder	1.0–100	Y = 0.733X−0.112	0.9945	1.25	5
40	Ciprofloxacin	Milk	1.0–100	Y = 2.749X + 0.514	0.9959	0.25	1
		Milk powder	1.0–100	Y = 2.322X−0.193	0.9991	1.25	5
41	Pefloxacin	Milk	1.0–100	Y = 1.693X + 0.305	0.9983	0.25	1
		Milk powder	1.0–100	Y = 1.548X−0.064	0.9951	1.25	5
42	Ofloxacin	Milk	1.0–100	Y = 2.534X + 0.455	0.9982	0.25	1
		Milk powder	1.0–100	Y = 2.572X−0.064	0.9956	1.25	5
43	Marbofloxacin	Milk	1.0–100	Y = 0.968X + 0.171	0.9987	0.25	1
		Milk powder	1.0–100	Y = 1.046X−0.053	0.9947	1.25	5
44	Flerofloxacin	Milk	1.0–100	Y = 1.430X + 0.230	0.9977	0.25	1
		Milk powder	1.0–100	Y = 1.750X−0.116	0.9950	1.25	5
45	Pipemidic acid	Milk	1.0–100	Y = 1.793X−0.257	0.9966	0.25	1
		Milk powder	1.0–100	Y = 1.809X + 0.238	0.9968	1.25	5
46	Oxolinic acid	Milk	1.0–100	Y = 5.431X + 1.046	0.9975	0.25	1
		Milk powder	1.0–100	Y = 5.536X + 0.109	0.9968	1.25	5
47	Gatifloxacin	Milk	1.0–100	Y = 0.703X + 0.114	0.9962	0.25	1
		Milk powder	1.0–100	Y = 0.761X−0.084	0.9957	1.25	5
48	Sarafloxacin	Milk	1.0–100	Y = 1.079X−0.073	0.9985	0.25	1
		Milk powder	1.0–100	Y = 1.102X + 0.159	0.9962	1.25	5
49	Sparfloxaxin	Milk	1.0–100	Y = 1.772X + 0.060	0.9990	0.25	1
		Milk powder	1.0–100	Y = 1.888X + 0.041	0.9983	1.25	5
50	Difloxaxin	Milk	1.0–100	Y = 2.449X + 0.488	0.9979	0.25	1
		Milk powder	1.0–100	Y = 2.081X + 0.037	0.9983	1.25	5
**22 Sulfonamides**							
51	Sulphanilamide	Milk	0.5–100	Y = 0.104X−0.003	0.9988	0.25	0.5
		Milk powder	0.5–100	Y = 0.153X−0.031	0.9966	1.25	2.5
52	Sulfaguanidine	Milk	1.0–100	Y = 0.034X−0.004	0.9990	0.5	1
		Milk powder	1.0–100	Y = 0.047X−0.019	0.9938	2.5	5
53	Sulfacetamide	Milk	0.5–100	Y = 0.026X−0.001	0.9987	0.25	0.5
		Milk powder	0.5–100	Y = 0.033X−0.010	0.9952	1.25	2.5
54	Sulfadiazine	Milk	0.5–100	Y = 0.754X−0.010	0.9994	0.25	0.5
		Milk powder	0.5–100	Y = 1.107X−0.380	0.9959	1.25	2.5
55	Sulfamethoxazole	Milk	0.5–50	Y = 0.643X + 0.020	0.9990	0.25	0.5
		Milk powder	0.5–50	Y = 0.903X−0.209	0.9974	1.25	2.5
56	Sulfamoxole	Milk	0.5–100	Y = 1.026X + 0.473	0.9977	0.25	0.5
		Milk powder	0.5–100	Y = 1.650X−0.512	0.9967	1.25	2.5
57	Sulfadoxine	Milk	0.25–50	Y = 1.282X−0.286	0.9969	0.25	0.25
		Milk powder	0.25–50	Y = 2.720X + 0.053	0.9988	1.25	1.25
58	Sulfamethizole	Milk	0.5–100	Y = 1.087X + 0.062	0.9975	0.25	0.5
		Milk powder	0.5–100	Y = 1.775X−0.573	0.9955	1.25	2.5
59	Sulfadimidine	Milk	0.5–100	Y = 1.083X + 0.096	0.9942	0.25	0.5
		Milk powder	0.5–100	Y = 1.976X−0.587	0.9957	1.25	2.5
60	Sulfameter	Milk	0.5–100	Y = 0.771X + 0.044	0.9980	0.25	0.5
		Milk powder	0.5–100	Y = 1.090X−0.308	0.9963	1.25	2.5
61	Sulfamethoxypyridazine	Milk	0.5–100	Y = 1.532X + 0.140	0.9942	0.25	0.5
		Milk powder	0.5–100	Y = 2.519X−0.595	0.9966	1.25	2.5
62	Sulfisoxazole	Milk	0.5–100	Y = 1.308X + 0.112	0.9950	0.25	0.5
		Milk powder	0.5–100	Y = 2.477X−0.671	0.9970	1.25	2.5
63	Sulfapyridine	Milk	0.5–100	Y = 1.457X + 0.162	0.9939	0.25	0.5
		Milk powder	0.5–100	Y = 2.353X−0.767	0.9961	1.25	2.5
64	Sulfathiazole	Milk	0.5–100	Y = 0.615X + 0.030	0.9991	0.25	0.5
65	Sulfamerazine	Milk	0.5–100	Y = 1.013X + 0.109	0.9946	0.25	0.5
		Milk powder	0.5–100	Y = 1.793X−0.517	0.9978	1.25	2.5
66	Trimethoprim	Milk	0.5–100	Y = 0.952X + 0.170	0.9917	0.25	0.5
		Milk powder	0.5–100	Y = 1.057X−0.650	0.9954	1.25	2.5
67	Sulfabenzamide	Milk	0.5–100	Y = 0.376X−0.010	0.9991	0.25	0.5
		Milk powder	0.5–100	Y = 0.475X−0.183	0.9955	1.25	2.5
68	Sulfaquinoxaline	Milk	0.5–100	Y = 0.715X + 0.002	0.9991	0.25	0.5
		Milk powder	0.5–100	Y = 0.937X−0.231	0.9977	1.25	2.5
69	Sulfadimethoxine	Milk	0.25–50	Y = 1.422X + 0.011	0.9981	0.25	0.25
		Milk powder	0.25–50	Y = 1.705X−0.241	0.9975	1.25	1.25
70	Sulfaphenazole	Milk	0.5–100	Y = 0.293X + 0.006	0.9980	0.25	0.5
		Milk powder	0.5–100	Y = 0.383X−0.071	0.9980	1.25	2.5
71	Sulfamonomethoxine	Milk	0.5–100	Y = 1.218X + 0.085	0.9965	0.25	0.5
		Milk powder	0.5–100	Y = 2.087X−0.573	0.9953	1.25	2.5
72	Sulfachloropyridazine	Milk	0.5–100	Y = 1.110X + 0.084	0.9962	0.25	0.5
		Milk powder	0.5–100	Y = 1.890X−0.493	0.9962	1.25	2.5
**8 Benzimidazoles**							
73	Albendazole sulfone^*^	Milk	0.5–50	Y = 8,1371X + 531.7	0.9972	0.5	1
		Milk powder	0.5–50	Y = 74,424X−710.1	0.9984	2.5	5
74	Oxfendazole^*^	Milk	0.5–50	Y = 89,002X + 1,207	0.9972	0.5	1
		Milk powder	0.5–50	Y = 79,036X + 1,958	0.9991	2.5	5
75	Albendazole S-oxide^*^	Milk	0.5–50	Y = 60,846X + 160.1	0.9974	0.5	1
		Milk powder	0.5–50	Y = 53,345X + 31.79	0.9984	2.5	5
76	Fenbendazole^*^	Milk	0.5–50	Y = 6,277X + 782.37	0.9952	0.5	1
		Milk powder	0.5–50	Y = 4,096X + 1,411.5	0.9937	2.5	5
77	Flubendazole^*^	Milk	0.5–50	Y = 53,388X + 1,303	0.9995	0.5	1
		Milk powder	0.5–50	Y = 38,169X + 1,464	0.9947	2.5	5
78	Fenbendazole sulfone^*^	Milk	0.5–50	Y = 57,550X + 396.3	0.9967	0.5	1
		Milk powder	0.5–50	Y = 4,257X + 106.2	0.9938	2.5	5
79	Mebendazole^*^	Milk	0.5–50	Y = 61,256X + 404.9	0.9969	0.5	1
		Milk powder	0.5–50	Y = 58,564X + 315.2	0.9952	2.5	5
80	Albendazole^*^	Milk	0.5–50	Y = 29,005X + 1,633	0.9965	0.5	1
		Milk powder	0.5–50	Y = 27,227X + 2,098	0.9961	2.5	5
**4 Exogenous hormones**							
81	Zeranol	Milk	1.0–50	Y = 14.45X + 3.849	0.9974	0.5	1
		Milk powder	5.0–250	Y = 14.75X + 5.401	0.9989	2.5	5
82	Hexestrol	Milk	1.0–50	Y = 0.426X + 0.082	0.9994	0.5	1
		Milk powder	5.0–250	Y = 0.526X−0.011	0.9985	2.5	5
83	Diethyatibestrol	Milk	1.0–50	Y = 1.141X + 0.443	0.9971	0.5	1
		Milk powder	5.0–250	Y = 1.278X−0.192	0.9996	2.5	5
84	Dienestrol	Milk	1.0–50	Y = 2.482X + 0.644	0.9986	0.3	1
		Milk powder	5.0–250	Y = 2.911X + 0.308	0.9988	1.5	5
**3 Chloramphenicol**							
85	Chloramphenicol	Milk	0.1–5.0	Y = 3.097X + 0.003	0.9937	0.05	0.1
		Milk powder	0.5–2.5	Y = 3.122X−0.064	0.9911	0.25	0.5
86	Thiamphenicol	Milk	1.0–100	Y = 0.727X + 0.833	0.9932	0.5	2
		Milk powder	5.0–500	Y = 1.081X + 0.501	0.9968	2.5	10
87	Florfeniol	Milk	1.0–100	Y = 2.784X + 0.788	0.9994	0.5	2
		Milk powder	5.0–500	Y = 3.437X + 1.753	0.9963	2.5	10
**8 glucocorticoids**							
88	Hydrocortisone	Milk	0.5–20	Y = 1.254X + 0.378	0.9902	0.2	0.5
		Milk powder	2.5–100	Y = 1.295X + 0.358	0.9979	1	2.5
89	Prednisolone	Milk	0.5–20	Y = 1.573X + 0.103	0.9933	0.2	0.5
		Milk powder	2.5–100	Y = 1.638X + 0.356	0.9971	1	2.5
90	Prednisone	Milk	0.5–20	Y = 0.340X−0.016	0.9926	0.2	0.5
		Milk powder	2.5–100	Y = 0.330X + 0.085	0.9909	1	2.5
91	Beclomethasone	Milk	0.5–20	Y = 6.838X + 1.531	0.9930	0.2	0.5
		Milk powder	2.5–100	Y = 6.925X + 2.035	0.9936	1	2.5
92	Betamethasone	Milk	0.5–20	Y = 1.653X−0.219	0.9970	0.2	0.5
		Milk powder	2.5–100	Y = 1.656X + 0.205	0.9923	1	2.5
93	Dexamethasone	Milk	0.5–20	Y = 1.959X + 0.261	0.9921	0.2	0.5
		Milk powder	2.5–100	Y = 1.814X + 0.128	0.9964	1	2.5
94	Fludrocortisone	Milk	0.5–20	Y = 21.89X + 2.653	0.9945	0.2	0.5
		Milk powder	2.5–100	Y = 23.11X + 5.674	0.9967	1	2.5
95	Methylprednisolone	Milk	0.5–20	Y = 2.476X + 0.328	0.9965	0.2	0.5
		Milk powder	2.5–100	Y = 2.245X + 0.369	0.9948	1	2.5
**4 Endogenous estrogens**							
96	Ethinylestradiol	Milk	5.0–200	Y = 2.711X−6.517	0.9971	2.5	10
		Milk powder	25.0–1,000	Y = 1.842X−0.683	0.9963	12.5	50
97	Estriol	Milk	5.0–200	Y = 10.86X−35.08	0.9987	1.5	5
		Milk powder	25.0–1,000	Y = 8.440X−6.388	0.9959	12.5	25
98	17-α-estradiol	Milk	5.0–200	Y = 7.897X−19.83	0.9993	1.5	5
		Milk powder	25.0–1,000	Y = 4.247X−1.891	0.9983	12.5	25
99	17-β-estradiol	Milk	5.0–200	Y = 1.860X−8.540	0.9907	2.5	10
		Milk powder	25.0–1,000	Y = 1.424X−3.214	0.9998	12.5	50
**4 Triazines**							
100	Toltrazuril	Milk	1.0–50	Y = 0.266X + 0.042	0.9994	0.3	1
		Milk powder	5.0–250	Y = 0.285X + 0.027	0.9998	1.5	5
101	Toltrazuril sulfoxide	Milk	1.0–50	Y = 0.104X−0.058	0.9966	0.3	1
		Milk powder	5.0–250	Y = 0.255X−0.008	0.9964	1.5	5
102	Toltrazuril sulfone	Milk	1.0–50	Y = 1.494X−0.213	0.9992	0.3	1
		Milk powder	5.0–250	Y = 0.906X + 0.080	0.9986	1.5	5
103	Diclazuril	Milk	1.0–50	Y = 0.528X + 0.999	0.9986	0.3	1
		Milk powder	5.0–250	Y = 0.816X + 0.014	0.9996	1.5	5

* *Used external standard method*.

#### Recovery and precision

The blank milk and milk powder samples were selected, and the recovery and precision experiments were carried out according to the above pretreatment methods. Recovery experiments were carried out at three levels, and six parallel tests were made for each level. The results showed that the recoveries in milk was from 31.1 to 118.8%, the relative standard deviations (intra-day RSD) ranged from 2.34 to 19.2%, and the recovery of milk powder was in the range of 42.9–120.7%, and the range of intra-day RSDs were 4.5–18.4%. Ampicillin achieved the lowest results of recovery of 37.1–50.2%, and estriol had the largest RSD of 19.2%. The recovery results of all drugs were shown in Table 6. The recovery of some drugs in this study needs to be improved compared with the detection method of a single or single kind of veterinary drug, but as a rapid screening method, it is applicable to the latest announcement issued by the Food and Drug Administration (Food and Drug Administration, FDA) on March 22, 2012: if the screening purpose of veterinary drug residue in food proves that the drug to be tested can be detected, The recovery rate can be relaxed to an appropriate extent (19).

**Table 6:** Recoveries and *RSD*s of analytes of milk and milk powder.

**No**.	**Analyte**	**Milk**				**Milk powder**			
		**Spiked** **(**μ**g/kg)**	**Average recovery** **(%)**	**Intra-day RSD, % (*****n*** = **6)**	**Inter-day RSD, %** **(*****n*** = **18)**	**Spiked** **(**μ**g/kg)**	**Average recovery** **(%)**	**Intra-day RSD, %** **(*****n*** = **6)**	**Inter-day RSD, %** **(*****n*** = **18)**
1	Dimetridazole	0.5/1/5	95.4/97.4/96.9	3.7/4.7/4.5	4.7/5.2/5.3	2.5/5/25	83.2/93.2/96.7	6.0/5.1/4.8	6.5/5.4/5.0
2	HMMNI	1/2/10	108.8/104.6/97.7	7.3/6.9/7.9	7.7/7.6/8.7	5/10/50	86.4/93.2/101.7	6.9/4.1/5.7	7.4/5.7/6.1
3	Metronidazole	0.5/1/5	107.9/103.7/105.7	7.1/6.9/5.6	7.8/7.7/6.3	2.5/5/25	92.3/94.3/98.2	3.7/2.9/3.1	5.6/3.1/4.3
4	Metronidazole-OH	1/2/10	97.3/98.7/103.4	6.8/6.1/5.4	7.6/6.8/6.2	5/10/50	95.2/99.7/101.3	7.4/4.6/5.7	8.3/5.4/4.6
5	Ronidazole	0.5/1/5	93.1/94.7/99.2	5.5/4.1/3.4	6.2/4.6/4.6	2.5/5/25	76.4/89.3/97.4	4.9/3.7/3.1	5.7/4.2/3.7
6	Ipronidazole	0.5/1/5	101.5/109.3/116.2	3.3/3.5/3.7	7.1/6.6/4.7	2.5/5/25	85.6/95.1/96.7	5.1/4.8/3.6	6.1/5.2/4.2
7	Ipronidazole-OH	0.5/1/5	100.6/102.4/105.9	2.5/3.6/4.2	3.8/5.2/4.3	2.5/5/25	88.3/97.3/98.9	6.8/4.8/5.2	8.1/6.2/5.6
8	Ornidazole	0.5/1/5	103.7/101.3/102.9	10.2/9.4/8.1	11.8/10.9/9.4	2.5/5/25	98.3/97.6/94.3	7.9/6.3/6.7	8.9/7.1/6.9
9	Tinidazole	0.5/1/5	100.6/105/107.6	7.7/7.0/7.2	9.3/7.6/6.8	2.5/5/25	86.3/96.3/92.6	8.3/6.4/6.3	9.3/6.9/6.8
10	Ceftiofur	10/20/50	63.9/57.4/68.3	7.0/6.0/5.5	9.7/6.9/7.1	50/100/200	68.6/59/74.2	9.2/8.3/8.7	9.4/9.2/9.6
11	Penicillin V	10/20/50	67.7/80.9/86.2	9.2/8.1/7.6	10.5/9.2/8.7	50/100/200	79.9/83.2/93.1	10.3/8.1/8	11.4/9.3/8.9
12	Oxacillin	10/20/50	71.4/82/92.6	9.2/6.0/4.7	10.5/6.8/7.8	50/100/200	74.5/89.3/94.2	8.9/8.0/6.3	9.9/9.1/7.3
13	Nafcillin	10/20/50	72.8/85.9/93.3	12.5/10.2/11.5	13.3/11.6/9.9	50/100/200	81.7/93.7/89.2	9.2/7.8/6.5	10.2/8.1/7.2
14	Cloxacillin	10/20/50	72.1/87.9/97.8	11.3/9.4/9.3	12.4/9.9/9.5	50/100/200	87.9/89.7/93.5	12.8/9.0/9.2	13.1/9.3/10.2
15	Dicioxacillin	10/20/50	70.7/83.1/94	12.5/9.6/9.2	14.2/9.7.2/9.4	50/100/200	88.9/78.9/98.6	14.9/12.8/8.1	16.1/13.8/8.7
16	Penicillin G	10/20/50	88.5/90/92.1	11.5/8.8/5.6	13.1/9.6/8.7	50/100/200	78.9/90.2/96.9	10.6/8.0/7.4	11.4/8.6/8.2
17	Ampicillin	10/20/50	37.1/46.5/50.2	11.3/7.9/6.9	12.1/8.4/7.7	50/100/200	46.7/55.2/52.1	10.3/8.4/8.3	11.1/9.3/9.2
18	Piperacillin	10/20/50	61/82.5/86.1	10.1/6.1/5.5	10.9/7.8/6.7	50/100/200	79.1/80.2/84.3	9.4/8.5/7.5	10.1/9.2/8.1
19	Clindamycin	1/5/10	89.4/92.2/96.7	4.8/3.8/3.4	6.5/5.5/4.3	5/25/50	84/96.2/94.7	6.7/4.4/5.2	7.2/4.7/5.6
20	Lincomycin	1/5/10	104.3/107.3/109.8	4.2/3.7/4.1	5,7/4.6/4.3	5/25/50	93.2/96/94.6	5.1/4.1/4.9	6.5/4.4/5.3
21	Tilmicosin	5/10/20	105.1/106/108.7	4.7/3.9/5.2	6.3/4.7/4.8	25/50/100	84.1/85.4/96.1	5.4/4.2/7.5	6.8/4.7/8.4
22	Josamycin	5/10/20	95.9/100.1/105.3	10.2/6.3/7.5	10.8/7.5/6.8	25/50/100	82.3/92.4/89.4	7.8/8.3/6.4	8.7/9.3/7.1
23	Tamulin	5/10/20	109.3/106.8/102.1	5.0/4.8/4.0	5.7/5.5/5.7	25/50/100	99/94.2/96.2	5.2/5.2/4.6	7.8/5.8/5.1
24	Oleandomycin	5/10/20	102.5/104.8/99.8	4.7/4.5/6.3	5.4/5.2/7.2	25/50/100	85.4/93.2/96.4	7.9/5.5/6.8	8.8/6.1/7.5
25	Erythromycin	5/10/20	95.1/92.7/94.2	9.2/4.2/4.1	10.5/4.8/4.7	25/50/100	79.1/84.8/86.7	11.8/10.4/8.2	12.9/11.4/9
26	Stereomycin	5/10/20	83.7/93.8/97	8.3/6.6/4.9	9.4/7.1/5.2	25/50/100	74.3/85.7/88.3	7.4/7.3/6.9	8.1/8/7.6
27	Roxithromycin	5/10/20	113.5/99.2/101.1	5.7/4.4/4.7	6.1/4.7/5.2	25/50/100	80.2/89.4/87.6	5.6/5.1/4.7	6.1/5.6/5.1
28	Tylosin	5/10/20	76.6/82.5/74.6	7.6/6.3/5.2	8.7/7.2/6.2	25/50/100	62.4/68.5/77.5	7.6/6.6/8.0	8.3/7.3/8.9
29	Azithromycin	5/10/20	90.4/98.6/109.8	10.6/9.0/9.2	12.2/10.3/10.6	25/50/100	84.6/86.4/98.4	8.8/7.5/7.6	9.8/8.3/8.0
30	Spriamycin	5/10/20	91.7/95.6/98.7	5.6/3.5/4.6	7.7/7.4/6.1	25/50/100	70.3/74.1/80.3	7.7/8.1/7.6	8.5/9.2/8.0
31	Cinoxacin	1/5/10	96.1/116.1/109.6	3.1/4.2/3.9	7.3/6.6/5.8	5/25/50	80.3/87.4/94.3	5.8/6.7/4.0	6.4/7.4/4.4
32	Lomefloxaxin	1/5/10	107.8/112.7/105	5.1/4.1/6.3	5.9/4.4/6.7	5/25/50	78.7/85.9/94.2	5.4/6.5/6.0	6.9/7.2/6.7
33	Danofloxacin	1/5/10	86.8/112/106.5	6.0/5.9.0/9.7	6.4/6.3/10.3	5/25/50	75.6/88.2/80.8	7.3/6.7/8.4	8.1/7.4/8.9
34	Enrofloxacin	1/5/10	107.6/110.1/118.8	3.6/3.0/4.3	4.8/3.2/4.5	5/25/50	96.4/99.1/99.5	6.4/4.3/5.1	6.8/4.5/5.4
35	Orbifloxacin	1/5/10	115.8/113/107.7	3.7/3.4/4.1	5.9/3.6/4.3	5/25/50	84.2/88.7/88.5	5.5/5.6/6.9	7.8/5.9/7.3
36	Nalidixic acid	1/5/10	102.1/114.5/115.6	4.8/3.4/4.1	6.1/3.6/4.3	5/25/50	94.7/98.3/99.4	5.7/6.5/7.6	6.3/6.9/6.7
37	Flumequine	1/5/10	85.2/88.4/93.3	3.6/3.3/4.9	5.8/3.5/5.2	5/25/50	77.6/85.7/85	8.0/7.1/7.2	8.5/7.5/6.8
38	Norfloxaxin	1/5/10	81.6/90.4/101.2	7.5/5.3/4.8	7.9/5.6/5.1	5/25/50	77.2/89.8/97.3	7.8/5.4/6.1	8.6/6.9/6.8
39	Enoxacin	1/5/10	53.4/62.3/67.7	6.1/5.9/5.7	7.9/6.6/6.4	5/25/50	54.2/66.6/65.3	7.5/5.7/6.2	8.3/6.3/6.4
40	Ciprofloxacin	1/5/10	115.3/105.6/103.9	8.0/5.8/5.3	9.3/6.5/6.6	5/25/50	89.4/97.4/99.7	10.3/8.9/7.4	11.2/9.7/8.1
41	Pefloxacin	1/5/10	94.5/99.8/93.3	5.2/3.6/4.7	5.8/4.4/5.3	5/25/50	83.7/94.2/89.3	8.5/5.6/6.0	9.3/6.1/6.5
42	Ofloxacin	1/5/10	79.3/87.2/99.2	5.3/4.8/5.0	7.4/5.5/5.7	5/25/50	78.2/85.5/98.4	6.9/5.9/6.0	7.5/6.4/6.5
43	Marbofloxacin	1/5/10	87.3/94.4/99.6	4.9/4.6/5.4	5.6/5.2/6.1	5/25/50	86.3/87.8/99.1	8.4/6.3/5.6	9.0/6.8/6.1
44	Flerofloxacin	1/5/10	100.7/104.9/110.7	3.6/5.0/5.4	6.1/5.7/6.1	5/25/50	90.1/95.3/96.9	8.1/6.2/6.0	8.8/6.5/6.5
45	Pipemidic acid	1/5/10	43.1/57.1/55.4	5.1/5.3/4.9	6.8/6.1/5.6	5/25/50	45.6/53.9/48.9	9.3/10.1/10.1	10.1/9.9/10.0
46	Oxolinic acid	1/5/10	112.4/106.3/112.5	4.8/4.0/3.5	5.9/4.5/4.0	5/25/50	106.2/102.9/104.3	5.6/5.9/5.2	6.7/6.4/5.8
47	Gatifloxacin	1/5/10	91.4/101.1/102.7	3.6/4.0/3.1	6.1/4.4/4.4	5/25/50	90.7/83.3/89.4	5.6/4.9/6.1	6.2/5.4/6.8
48	Sarafloxacin	1/5/10	92.7/98.8/105.5	4.0/4.6/5.2	4.4/5.1/5.7	5/25/50	75.7/85.7/83.8	4.6/3.4/5.8	6.5/4.5/6.2
49	Sparfloxaxin	1/5/10	92.2/95.7/93.8	5.6/5.6/4.8	6.2/6.2/5.3	5/25/50	86.8/93.1/92	4.6/5.2/4.4	4.9/5.6/4.7
50	Difloxaxin	1/5/10	104.2/101.8/103.4	4.5/4.1/3.5	4.9/4.5/3.8	5/25/50	83.3/97/99.1	4.4/4.6/3.5	4.7/4.9/4.6
51	Sulphanilamide	1/5/10	108.3/102.7/105.5	7.8/5.5/5.1	8.6/6.3/5.7	5/25/50	87.6/92.7/96.7	7.1/6.8/7.4	7.6/7.4/7.9
52	Sulfaguanidine	1/5/10	97.8/103.7/114.6	5.4/5.5/4.7	7.8/6.1/5.2	5/25/50	92.6/96.8/100.4	5.2/5.7/6.1	5.8/6.3/6.8
53	Sulfacetamide	1/5/10	100.4/101.2/93.6	5.8/6.9/5.7	6.5/7.7/6.3	5/25/50	85.2/91.1/95.1	6.9/5.8/5.8	7.6/6.4/6.8
54	Sulfadiazine	1/5/10	91.5/95.1/97.7	6.7/5.8/5.4	7.5/6.5/6.3	5/25/50	97.2/108.2/98.9	7.8/7.9/6.1	9.6/8.8/7.8
55	Sulfamethoxazole	1/5/10	102.1/106.5/103.1	4.2/4.0/3.7	7.7/4.5/4.2	5/25/50	99.4/101.5/105.5	6.0/5.3/5.3	6.6/5.9/4.9
56	Sulfamoxole	1/5/10	103.5/106.3/109.1	4.8/3.9/3.4	5.4/4.4/3.9	5/25/50	89.9/98.3/103.5	4.6/8.4/7.3	5.1/9.3/8.1
57	Sulfadoxine	1/5/10	96.5/100.6/111.2	5.7/4.6/5.1	6.5/5.2/5.8	5/25/50	97.4/100.7/107.3	8.7/4.7/4.7	9.6/6.7/5.2
58	Sulfamethizole	1/5/10	96.8/90.2/100.5	6.5/5.4/4.6	7.4/6.1/5.2	5/25/50	98.5/105.6/104.6	4.3/5.3/5.9	4.8/5.9/6.5
59	Sulfadimidine	1/5/10	101.3/108.2/102.7	5.3/5.2/4.8	5.8/5.7/5.3	5/25/50	101.6/108.5/111.1	6.1/5.5/4.5	6.8/6.1/6.1
60	Sulfameter	1/5/10	101.7/109.2/107.1	4.1/3.2/3.3	4.5/3.8/3.6	5/25/50	91.6/98.6/101.1	6.1/5.4/6.9	7.8/6.4/7.9
61	Sulfamethoxypyridazine	1/5/10	98.1/100.3/94.5	6.0/5.3/5.4	6.6/6.3/5.9	5/25/50	100.9/101.1/101.9	5.3/4.8/6.0	6.1/5.5/6.9
62	Sulfisoxazole	1/5/10	93.1/101.4/96.5	6.1/7.2/6.0	6.7/7.9/5.6	5/25/50	98.9/120.7/115.9	6.4/7.5/5.7	7.3/8.6/6.5
63	Sulfapyridine	1/5/10	99.2/102.8/110.1	4.0/2.3/2.9	4.4/2.8/3.5	5/25/50	101.1/107.8/117	4.8/5.3/5.4	5.5/6.1/6.2
64	Sulfathiazole	1/5/10	106.6/109.4/100.9	6.4/5.2/4.1	7.7/6.2/4.9	5/25/50	99.3/110.1/115.6	5.4/4.8/4.8	7.2/5.5/5.3
65	Sulfamerazine	1/5/10	103.5/103.9/101.1	11.3/11.0/10.6	13.5/12.2/11.1	5/25/50	105.3/114.7/115.4	10.5/12/9.9	11.6/13.5/11.1
66	Trimethoprim	1/5/10	99.4/98.9/101.6	5.6/4.9/4.5	5.9/5.1/4.7	5/25/50	102.5/116.4/117.7	3.6/4.8/5.2	4.9/5.4/5.8
67	Sulfabenzamide	1/5/10	97.9/102.2/105.8	7.2/5.8/5.7	7.6/6.1/6.4	5/25/50	96.1/92.9/104.3	6.2/5.9/5.8	7.9/6.8/6.5
68	Sulfaquinoxaline	1/5/10	101/100.4/96	4.6/6.3/6.4	4.8/6.6/6.7	5/25/50	89.1/97.4/105.1	6.6/6.2/6.8	7.4/7.0/7.6
69	Sulfadimethoxine	1/5/10	108.3/103.1/105.4	5.8/5.8/6.3	7.1/6.3/6.6	5/25/50	99/105.3/115.3	6.1/5.6/6.8	6.8/6.3/8.3
70	Sulfaphenazole	1/5/10	104.1/95.3/101.2	6.7/7.1/6.2	7.3/7.6/7.4	5/25/50	94.4/91.2/100.3	8.2/7.7/7.8	9.2/8.6/7.4
71	Sulfamonomethoxine	1/5/10	99.6/94.9/98.5	5.1/4.8/4.1	5.4/5.1/4.4	5/25/50	90.5/92.6/101.9	5.2/4.5/7.0	5.8/5.5/7.8
72	Sulfachloropyridazine	1/5/10	100.6/102.8/105.8	6.4/6.3/8.5	7.8/6.7/9.1	5/25/50	97.3/97.7/101	7.4/6.8/6.0	8.3/7.3/6.7
73	Albendazole sulfone	1/5/10	103.8/116.8/117.3	6.1/6.3/5.5	8.5/6.7/5.9	5/25/50	92.3/98.8/102.4	6.5/5.9/3.8	7.3/6.6/5.3
74	Oxfendazole	1/5/10	89.8/91.5/93.7	7.2/7.0/6.0	7.9/7.5/6.4	5/25/50	86.1/96.3/93.4	5.6/5.7/6.1	7.3/6.4/6.8
75	Albendazole S-oxide	1/5/10	102.2/107.3/105	10.7/10.8/8.4	11.2/11.3/8.8	5/25/50	94.7/102.2/101.2	8.1/7.1/7.8	9.3/8.1/8.9
76	Fenbendazole	1/5/10	97.3/104.8/105.1	9.4/10.1/9.7	9.8/10.6/10.2	5/25/50	103.8/95.4/94	7.5/7.6/5.5	8.5/8.6/6.2
77	Flubendazole	1/5/10	103.8/105.4/102.9	5.6/4.2/4.1	5.9/4.4/4.3	5/25/50	95.1/97.6/101.2	6.7/6.5/5.5	7.6/7.4/6.0
78	Fenbendazole sulfone	1/5/10	100.4/104.4/102.7	8.1/7.2/7.3	8.5/7.5/7.6	5/25/50	94.3/92.3/91.3	9.8/6.8/6.7	11.1/7.8/7.7
79	Mebendazole	1/5/10	97.4/113.4/107.7	11.1/7.2/7.1	11.6/7.5/6.4	5/25/50	101.5/89.5/97.6	7.5/6.5/6.5	8.6/8.0/7.5
80	Albendazole	1/5/10	99.4/98.3/97.8	6.1/4.3/4.7	6.5/4.6/5.3	5/25/50	93.2/82.5/92.1	8.2/7.2/7.9	9.4/8.2/9.0
81	Zeranol	1/5/10	73.7/89/83.1	14.5/8.3/8.1	15.5/8.9/8.7	5/25/50	79.3/85.4/87.2	11.3/10.2/11.2	12.9/11.7/10.8
82	Hexestrol	1/5/10	57.6/89.4/92.6	15.5/13.7/12.9	16.6/14.6/13.8	5/25/50	53.1/86.5/103.7	13.2/10.4/9.5	15.1/110/10.9
83	Diethyatibestrol	1/5/10	61.7/77.1/83.8	11.3/7.4/5.8	12.1/7.9/6.2	5/25/50	62.1/74.7/89.8	12.3/8.4/6.9	14.1/9.6/8.5
84	Dienestrol	1/5/10	75.8/84.2/101.1	11.2/4.8/8.5	10.9/5.2/9.2	5/25/50	79.1/86.5/97.7	13.6/6.7/11.0	14.5/12.4/10.9
85	Chloramphenicol	0.2/1/5	95.9/95.3/91.5	8.1/10.4/4.3	8.8/11.3/4.7	1/5/25	90.2/92.6/92.3	10.7/11.4/5.2	13.2/12.8/8.9
86	Thiamphenicol	2/5/20	64.4/91/106.1	6.9/3.6/5.9	7.5/3.9/6.4	10/25/100	61.8/95.1/104.4	7.8/4.8/6.1	8.9/5.5/6.2
87	Florfeniol	2/5/20	83.4/90.3/110.4	4.9/5.2/7.1	5.3/5.7/7.7	10/25/100	82.7/89.1/104.1	7.6/4.8/5.2	8.0/6.5/5.9
88	Hydrocortisone	1/5/10	78.3/96.5/85.4	10.4/9.4/5.3	11.3/10.2/5.8	5/25/50	75.1/95.3/89.7	10.9/9.8/6.7	12.5/11.2/7.7
89	Prednisolone	1/5/10	61/96.9/83.9	10.9/5.2/14.1	11.8/9.7/15.3	5/25/50	64.1/98.3/87.1	9.4/7.2/15.0	10.7/8.2/7.8
90	Prednisone	1/5/10	90.1/96.1/83.9	11.8/12.8/14.1	12.8/13.9/10.3	5/25/50	87.9/94/89.8	9.5/13.2/11.9	13.5/13.4/12.6
91	Beclomethasone	1/5/10	86.3/108.1/99.7	12.5/7.8/8.7	13.6/8.5/9.5	5/25/50	82.5/110.5/98.1	10.3/8.2/8.0	11.8/9.4/9.1
92	Betamethasone	1/5/10	79.1/104.4/69.8	15.3/8.4/9.4	16.6/9.1/10.2	5/25/50	65.8/101.2/68.2	8.9/9.2/8.3	11.3/10.5/8.5
93	Dexamethasone	1/5/10	78.8/95.5/91.3	9.6/5.7/4.1	10.4/6.2/5.6	5/25/50	81.2/99.3/87.3	9.1/6.0/4.5	10.4/7.4/6.5
94	Fludrocortisone	1/5/10	72.2/100.7/97.1	6.8/5.7/14.6	7.4/6.2/10.2	5/25/50	76.3/97.9/98.6	9.8/6.2/12.2	11.2/7.1/7.9
95	Methylprednisolone	1/5/10	72.3/102.3/96	13.0/6.3/9.7	14.1/6.8/10.5	5/25/50	71.1/101/74.7	14.8/7.1/8.6	15.2/8.1/8.5
96	Ethinylestradiol	10/20/50	60.8/62.6/71.6	10.1/7.2/11.9	11.5/7.8/12.9	50/100/250	61.8/64.3/68.9	13.0/8.0/10.4	13.9/9.1/7.9
97	Estriol	5/10/50	49.8/44.5/73.6	19.1/19.2/11.8	19.5/18.6/12.1	25/50/250	54.1/42.9/71.4	10.3/18.4/11.0	11.8/12.5/12.3
98	17-α-estradiol	5/10/50	64.5/65.1/62.3	17.8/11.6/9.4	19.3/12.6/10.2	25/50/250	67.7/67.8/63.3	15.4/10.5/8.5	17.6/11.8/9.5
99	17-β-estradiol	10/20/50	90.3/72.3/73.7	11.2/14.4/7.1	12.1/15.5/8.8	50/100/250	86.8/68.2/73.4	14.1/12.2/8.5	15.8/13.9/9.2
100	Toltrazuril	1/2/10	83.9/93/98.4	7.9/10.0/3.2	8.5/10.8/9.8	5/10/50	78.8/101.3/97.6	6.8/8.7/5.2	9.8/9.9/6.7
101	Toltrazuril sulfoxide	1/2/10	107.2/97.9/78.6	8.0/3.5/7.9	8.3/7.6/8.6	5/10/50	102.7/92/76.6	8.4/6.2/8.2	9.6/7.4/8.6
102	Toltrazuril sulfone	1/2/10	83.3/94.3/77.8	4.7/8.1/6.8	5.3/8.6/7.3	5/10/50	78.8/90.2/70.9	6.8/8.4/5.4	7.8/9.3/7.6
103	Diclazuril	1/2/10	105.1/99.1/74.1	2.7/11.4/8.1	9.8/10.4/8.7	5/10/50	102.3/87.2/72.4	5.2/6.3/5.1	8.7/8.4/8.6

### Analysis of market samples

Twenty cow Milk Samples and 10 Milk Powder Samples Obtained From Local Supermarkets Were Analyzed. The Analysis Was Completed in Just 2 Days. And Lincomycin Was Found 10.2 ± 1.5 μg/kg in Only one Milk Sample, but the Concentration Was Below the Current MRL (0.15 ppm).

## Conclusions

A method based on QuEChERS and UPLC-MS/MS for the determination of common veterinary drug residues was investigated. One hundred three analytes were quantified and validated using selected daughter ions under optimized MRM condition with ESI (+) or ESI (–) mode. The clean-up with QuEChERS consist of C18, and anhydrous sodium sulfate was suitable for simultaneous analysis of multi-class in milk and milk powder. The method is accurate, simple, rapid, and economic, and can be applied as a screening method in the determination of drug residues in milk and dairy products.

## Data availability statement

The original contributions presented in the study are included in the article/supplementary material, further inquiries can be directed to the corresponding author/s.

## Author contributions

LFA and YZ conceived and designed the experiments. XJG, HT, and FY performed the experiments. SFF and JWZ analyzed the data. JMM wrote the original draft. All authors have read and approved the manuscript.

## Funding

This work was supported by the Science and Technology project of Hebei Province (21475501D) and Science and Technology project of general Administration of China (2019HK112).

## Conflict of interest

The authors declare that the research was conducted in the absence of any commercial or financial relationships that could be construed as a potential conflict of interest.

## Publisher's note

All claims expressed in this article are solely those of the authors and do not necessarily represent those of their affiliated organizations, or those of the publisher, the editors and the reviewers. Any product that may be evaluated in this article, or claim that may be made by its manufacturer, is not guaranteed or endorsed by the publisher.
