# Supplementary figures and images for: Rapid determination of 103 common veterinary drug residues in milk and dairy products by ultra performance liquid chromatography tandem mass spectrometry
Source: Front Nutr. 2022 Jul 22;9:879518. doi: 10.3389/fnut.2022.879518 (PMC9354588; doi:10.3389/fnut.2022.879518)

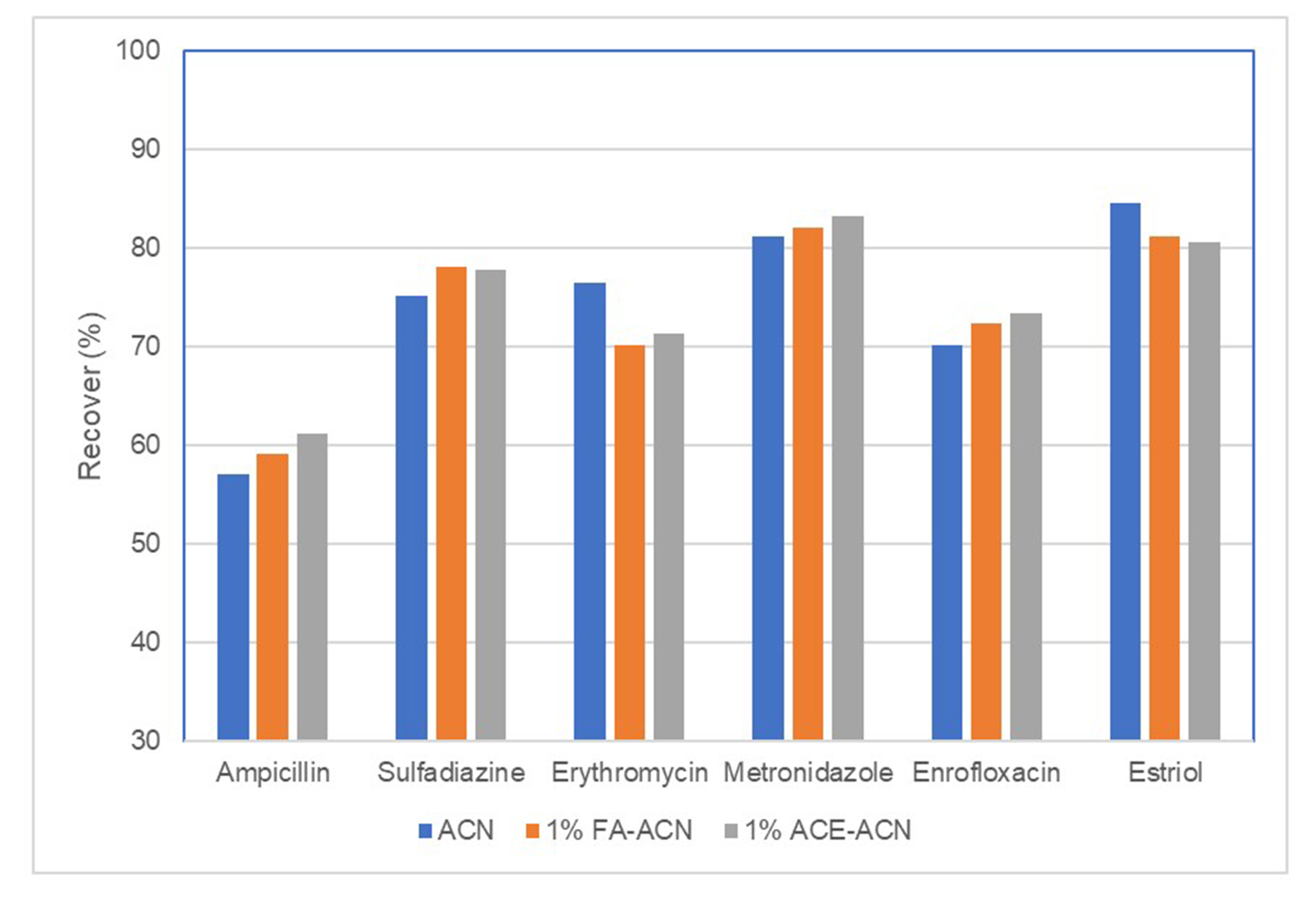

Supplement: Supplementary file 1 [file Image_1.jpg]

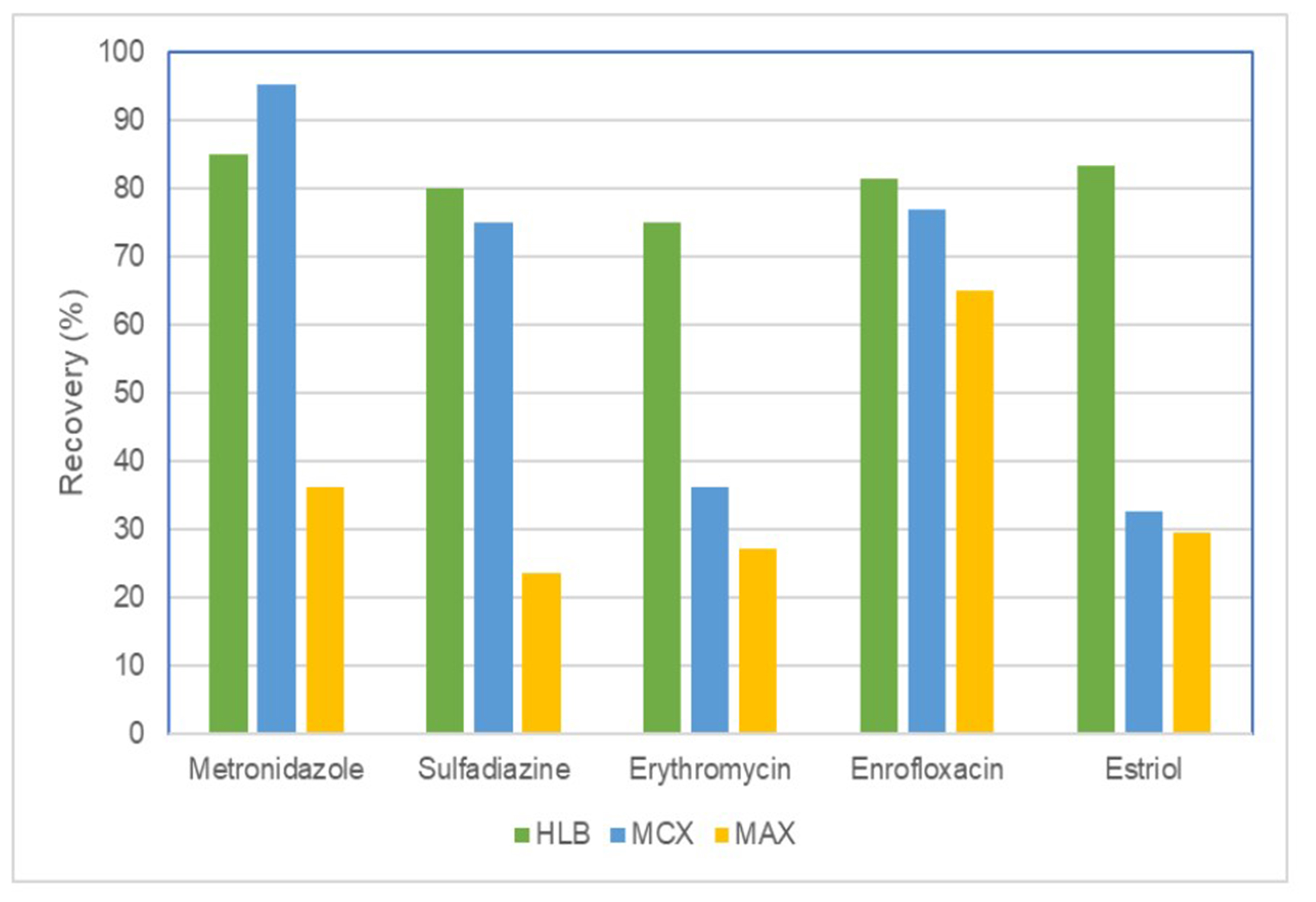

Supplement: Supplementary file 2 [file Image_2.jpg]

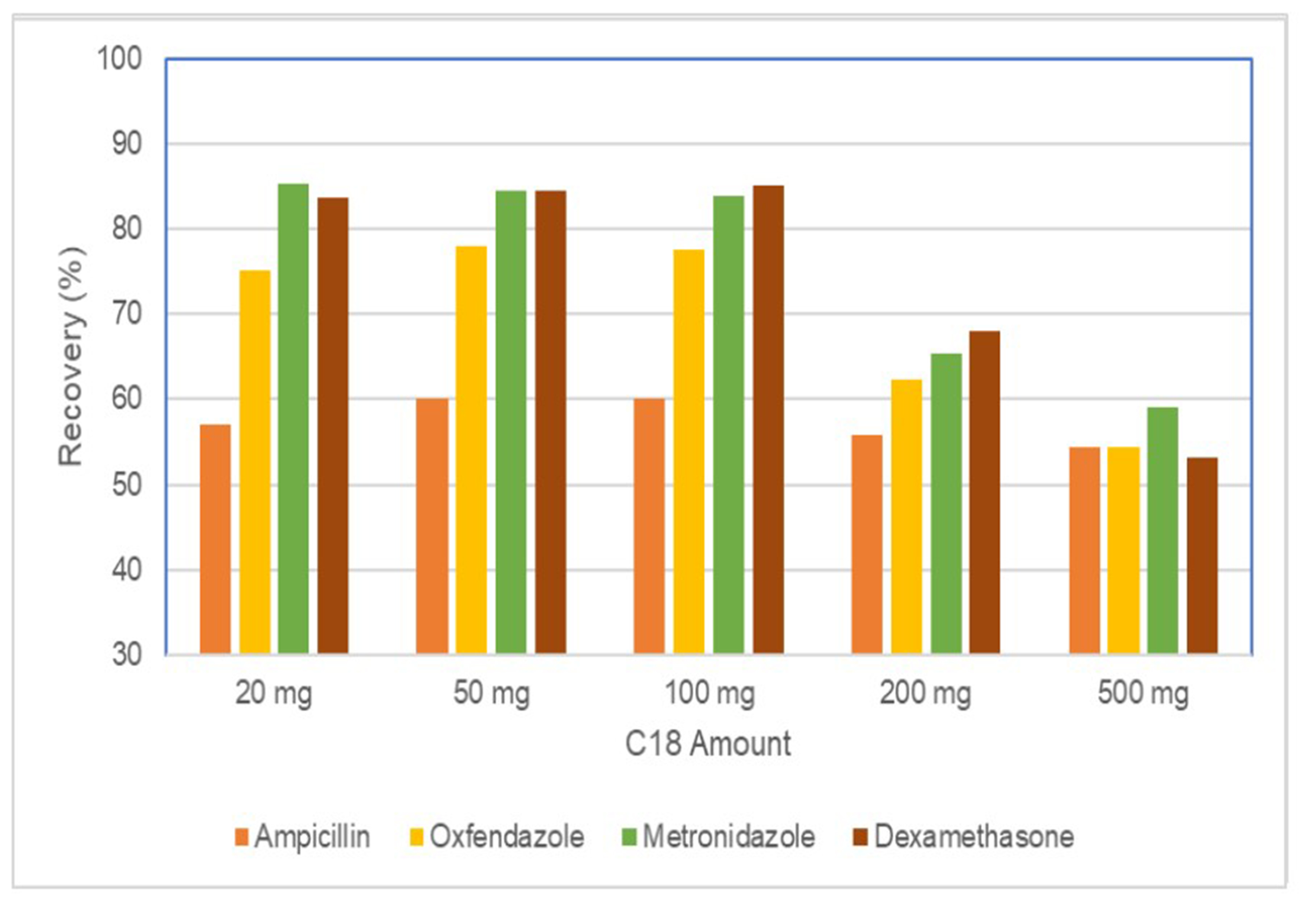

Supplement: Supplementary file 3 [file Image_3.jpg]

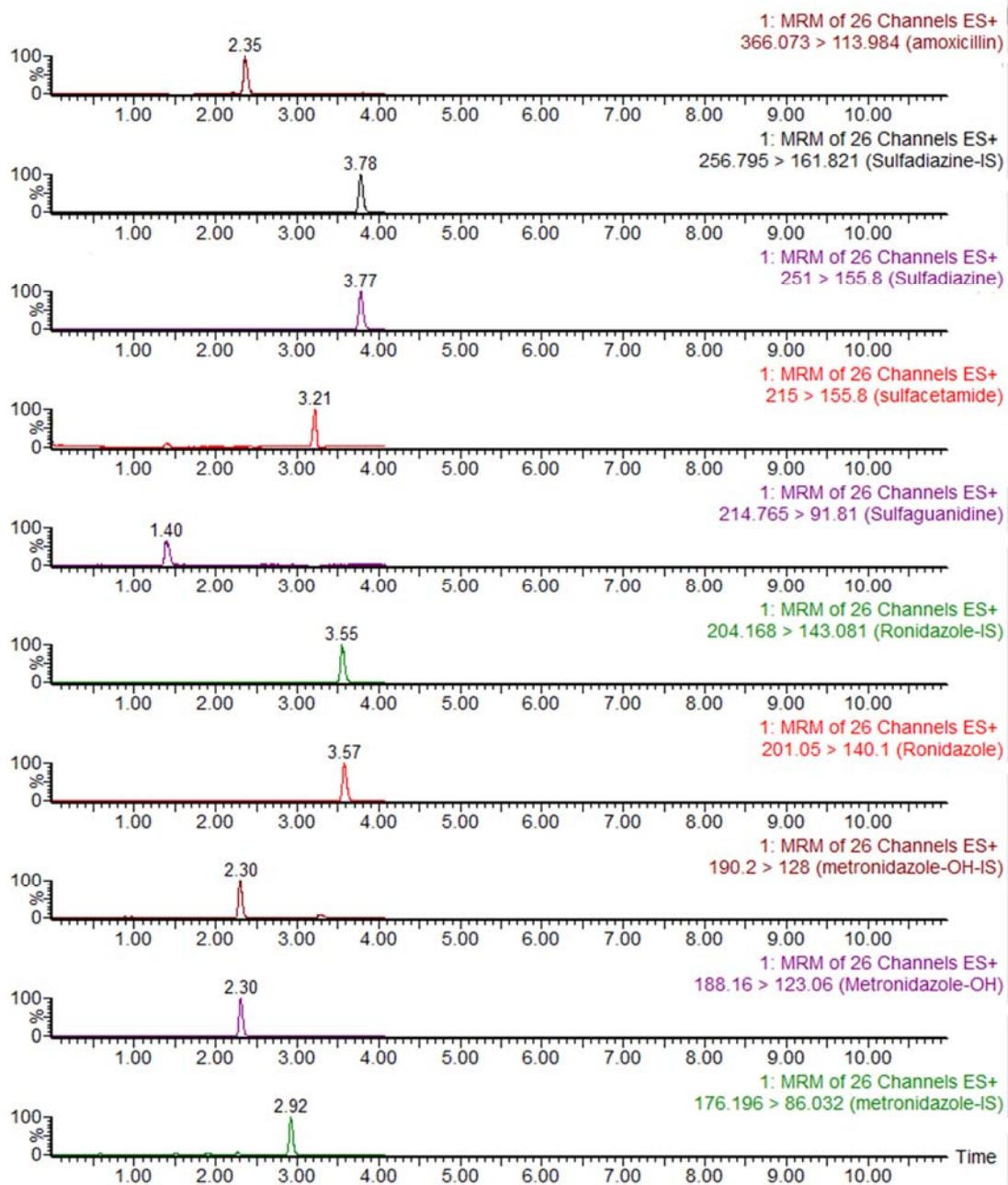

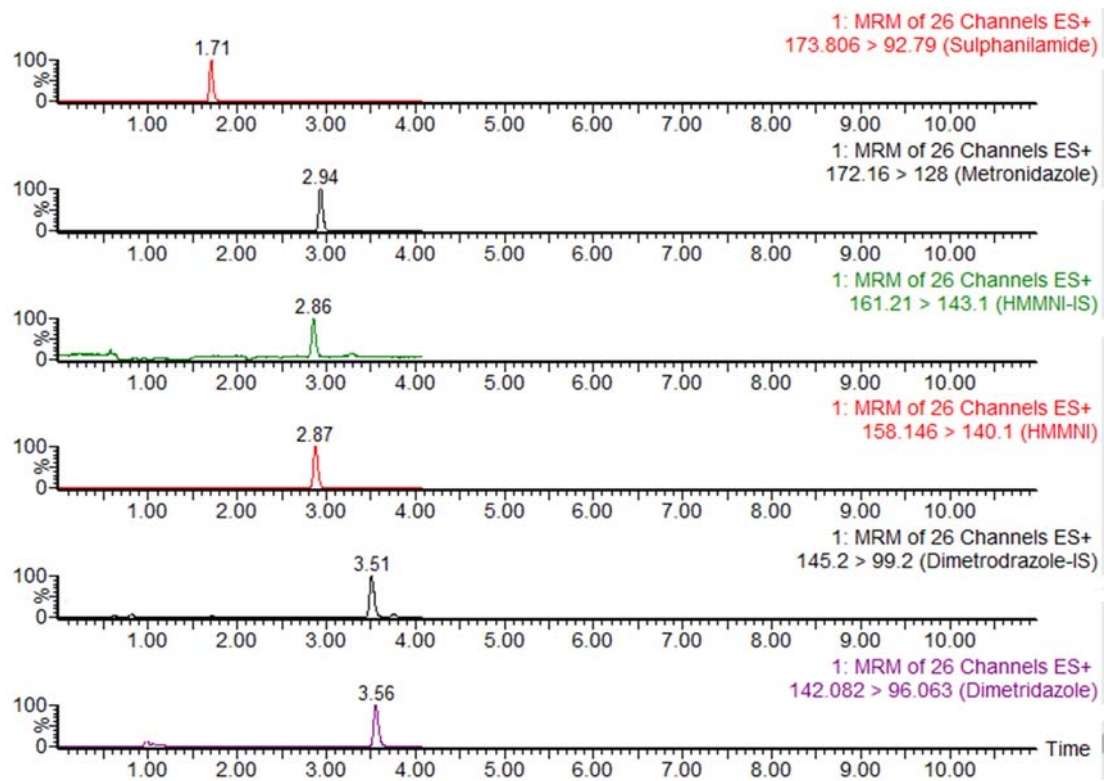

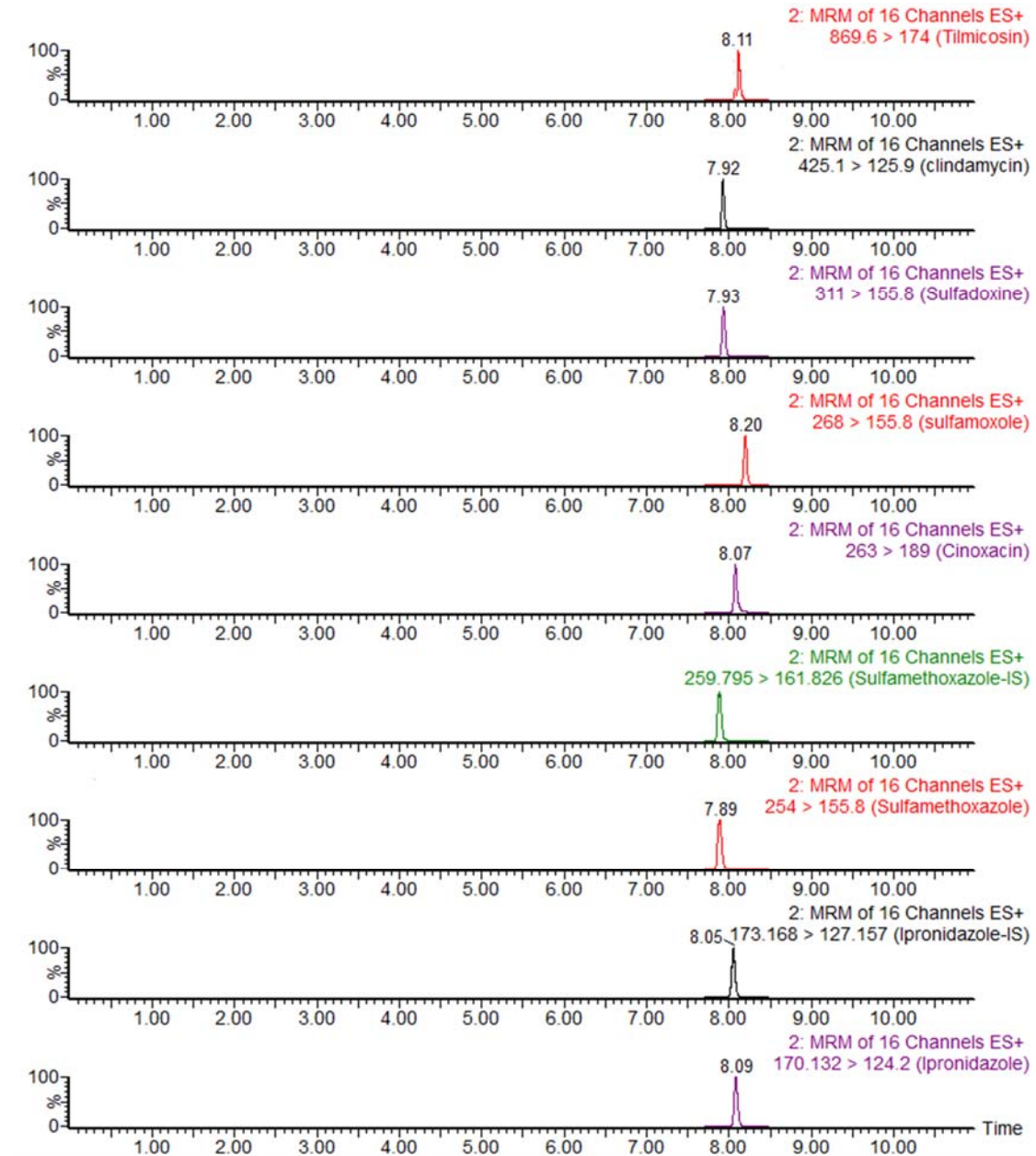

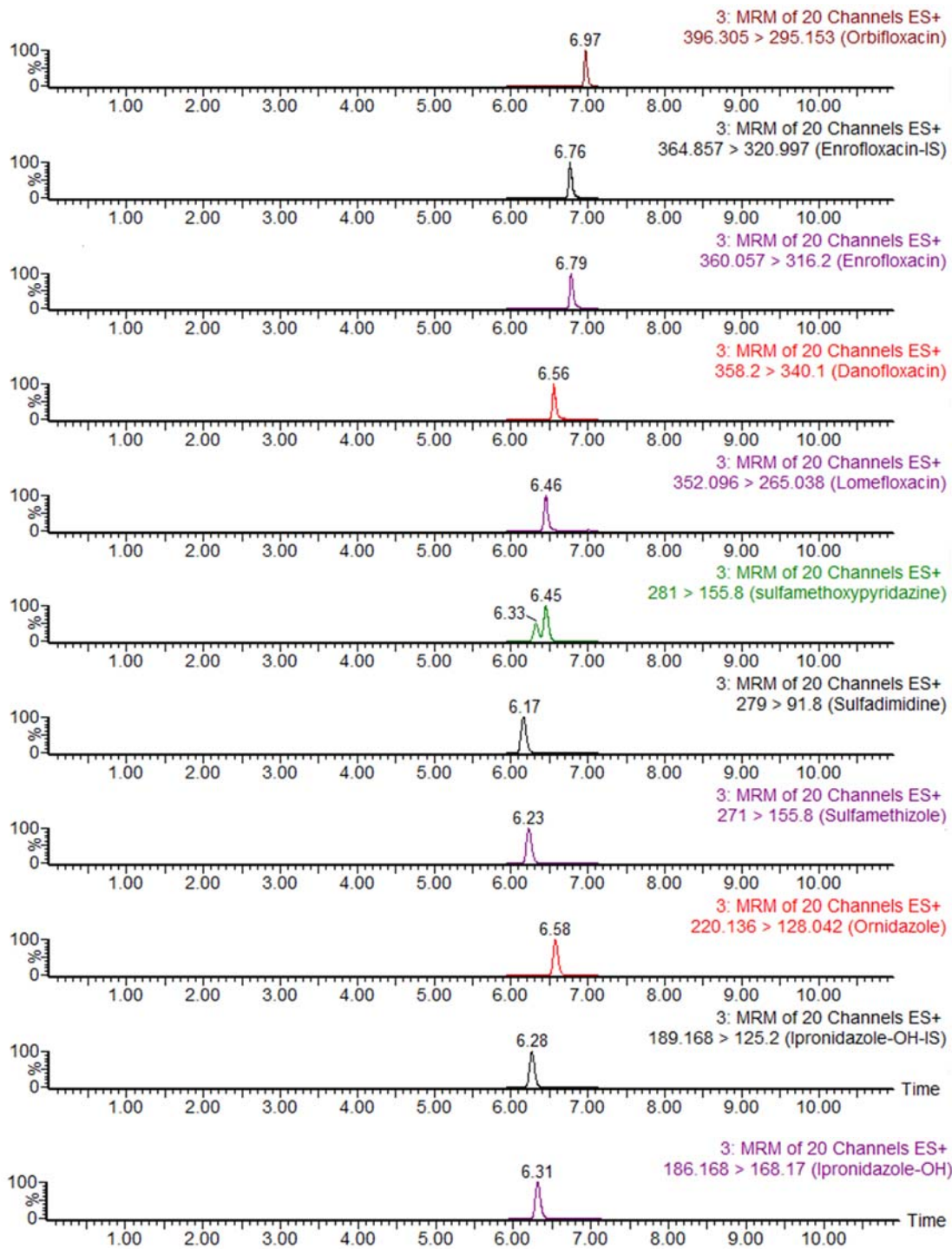

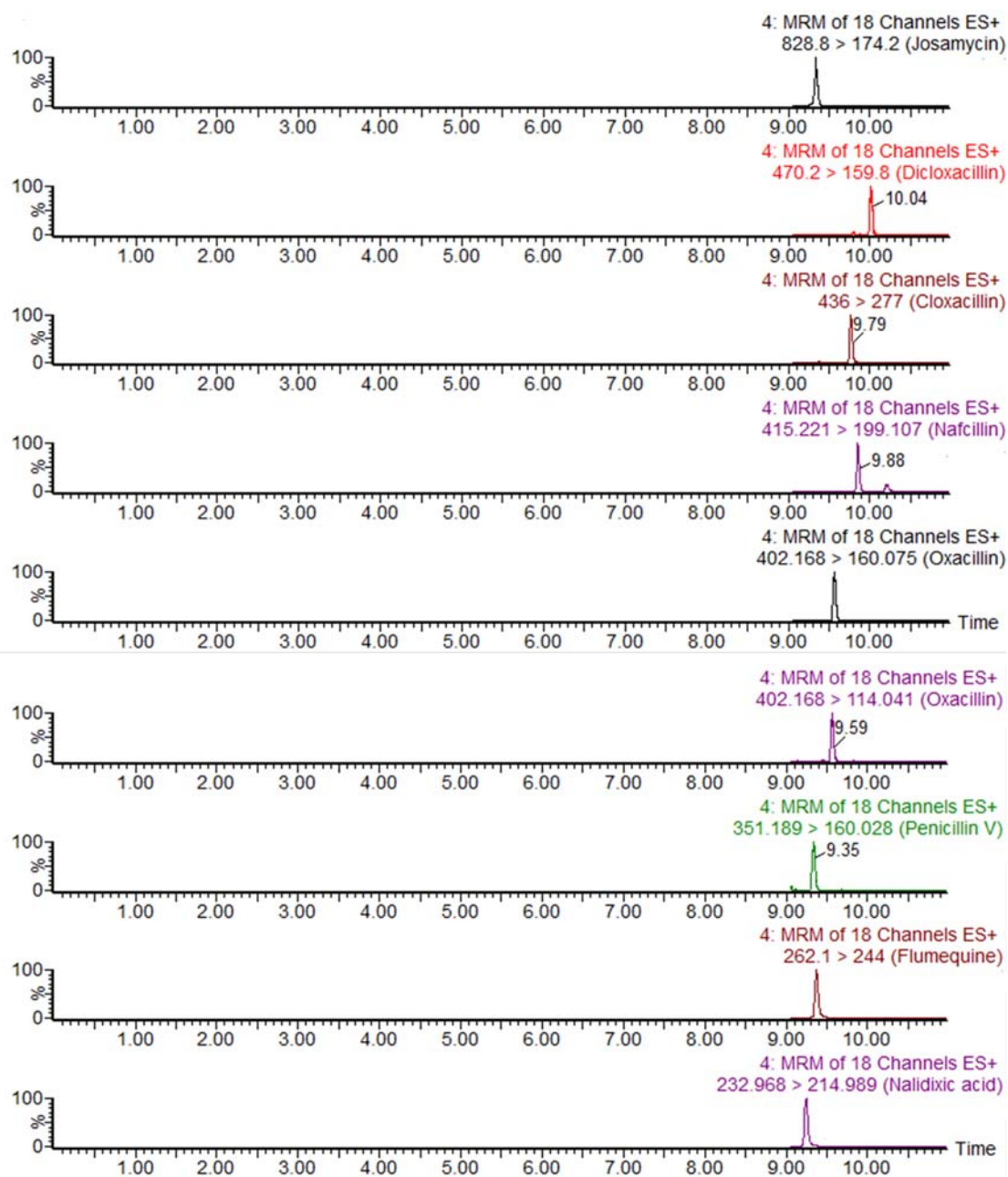

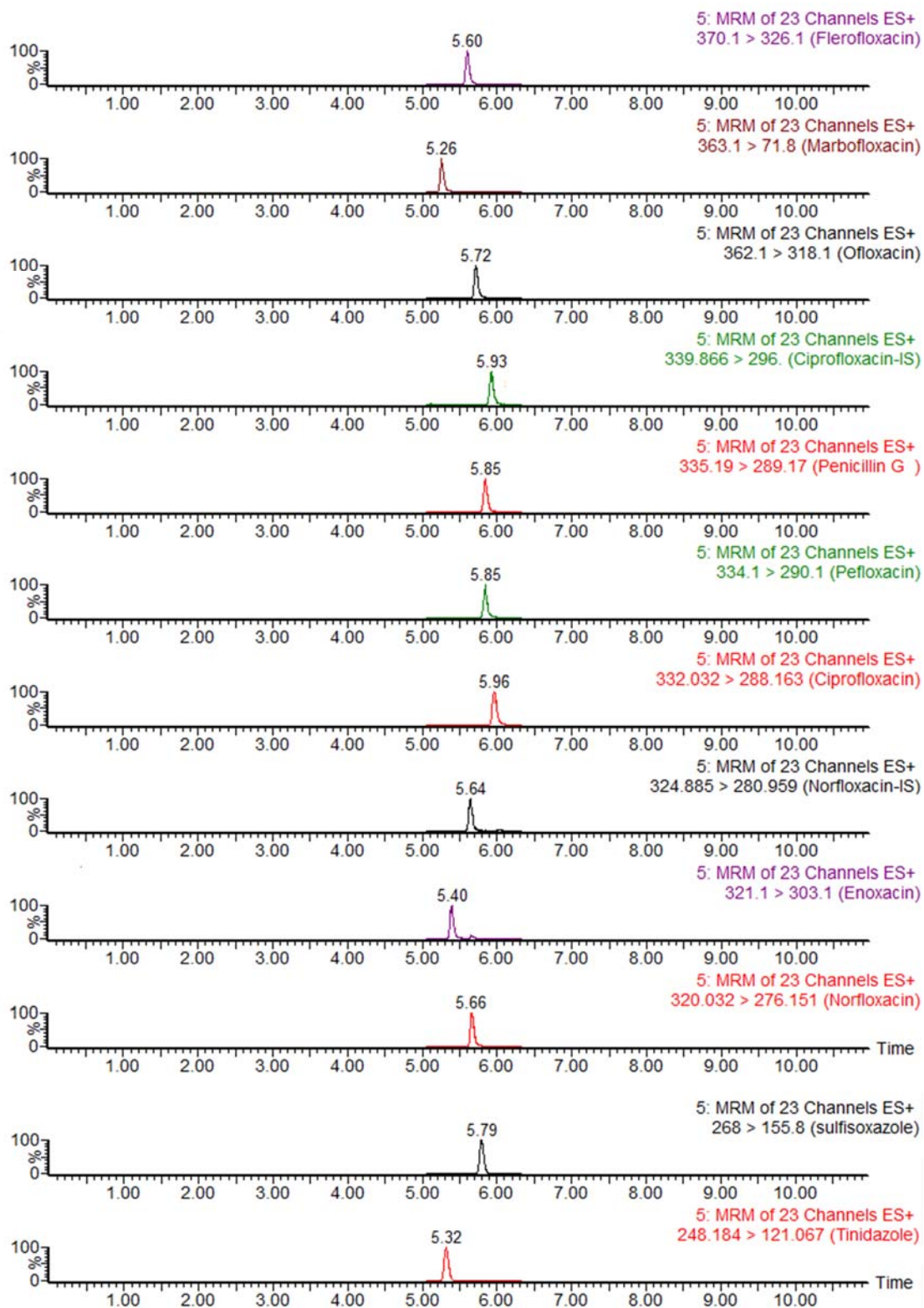

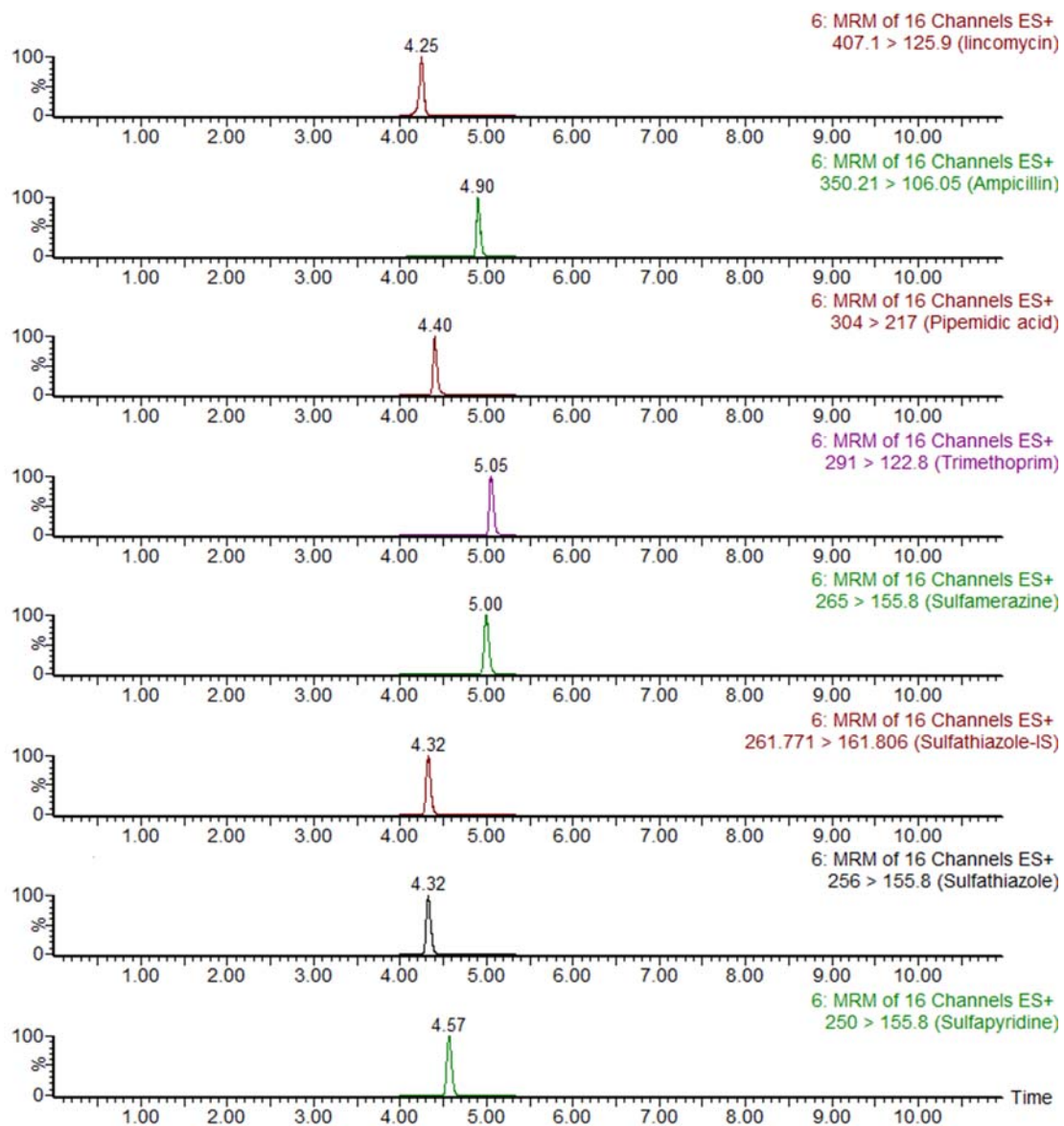

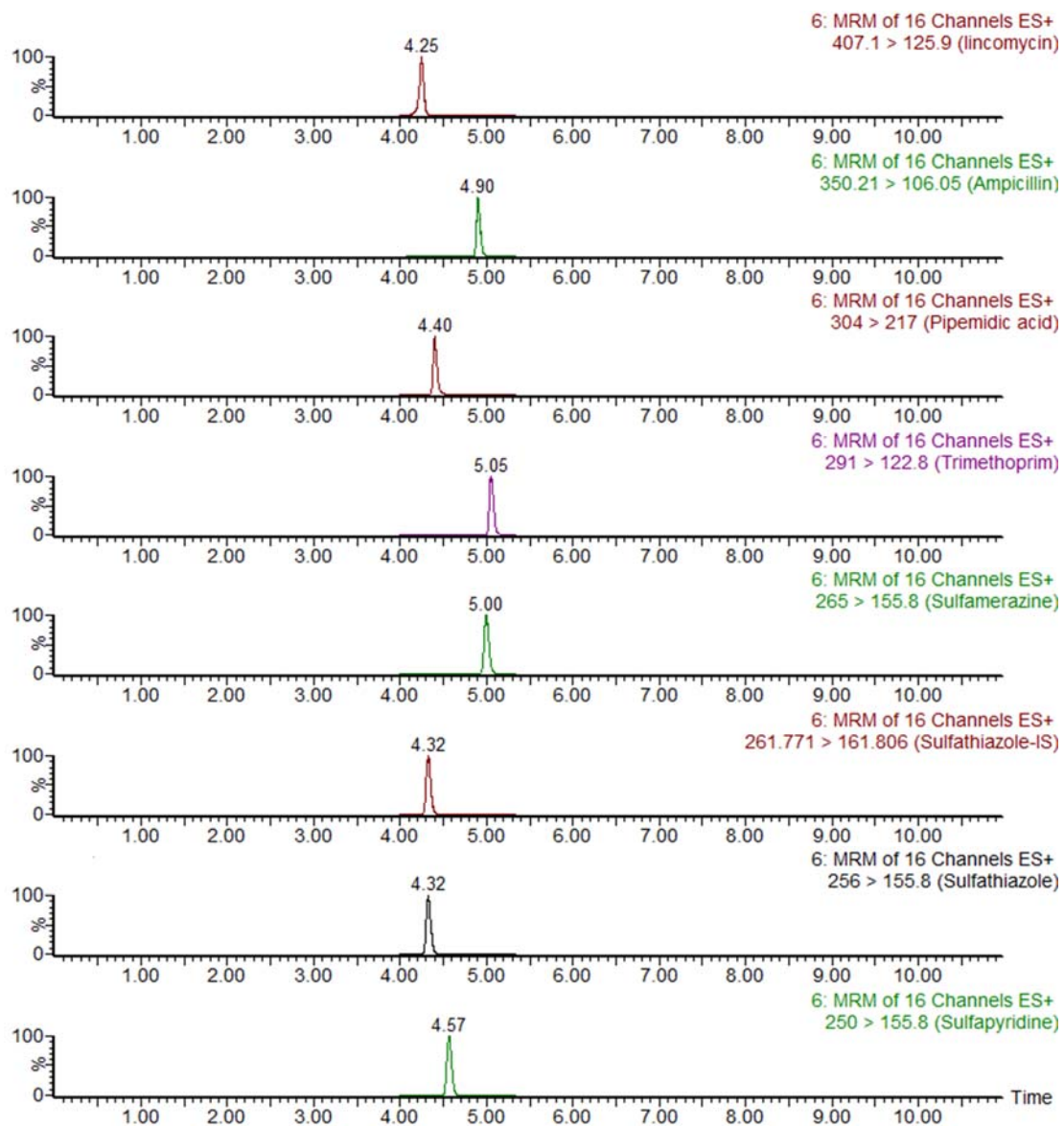

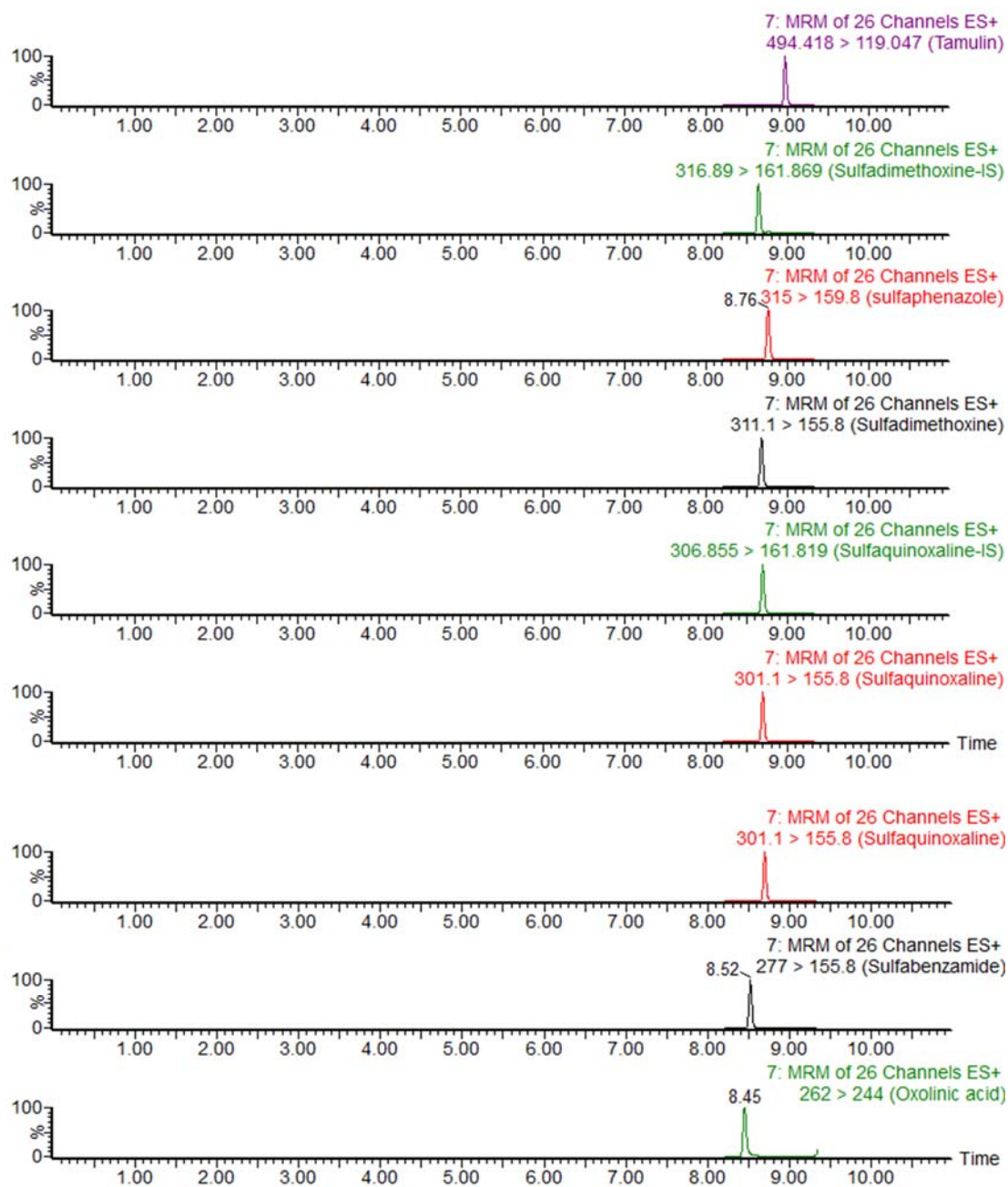

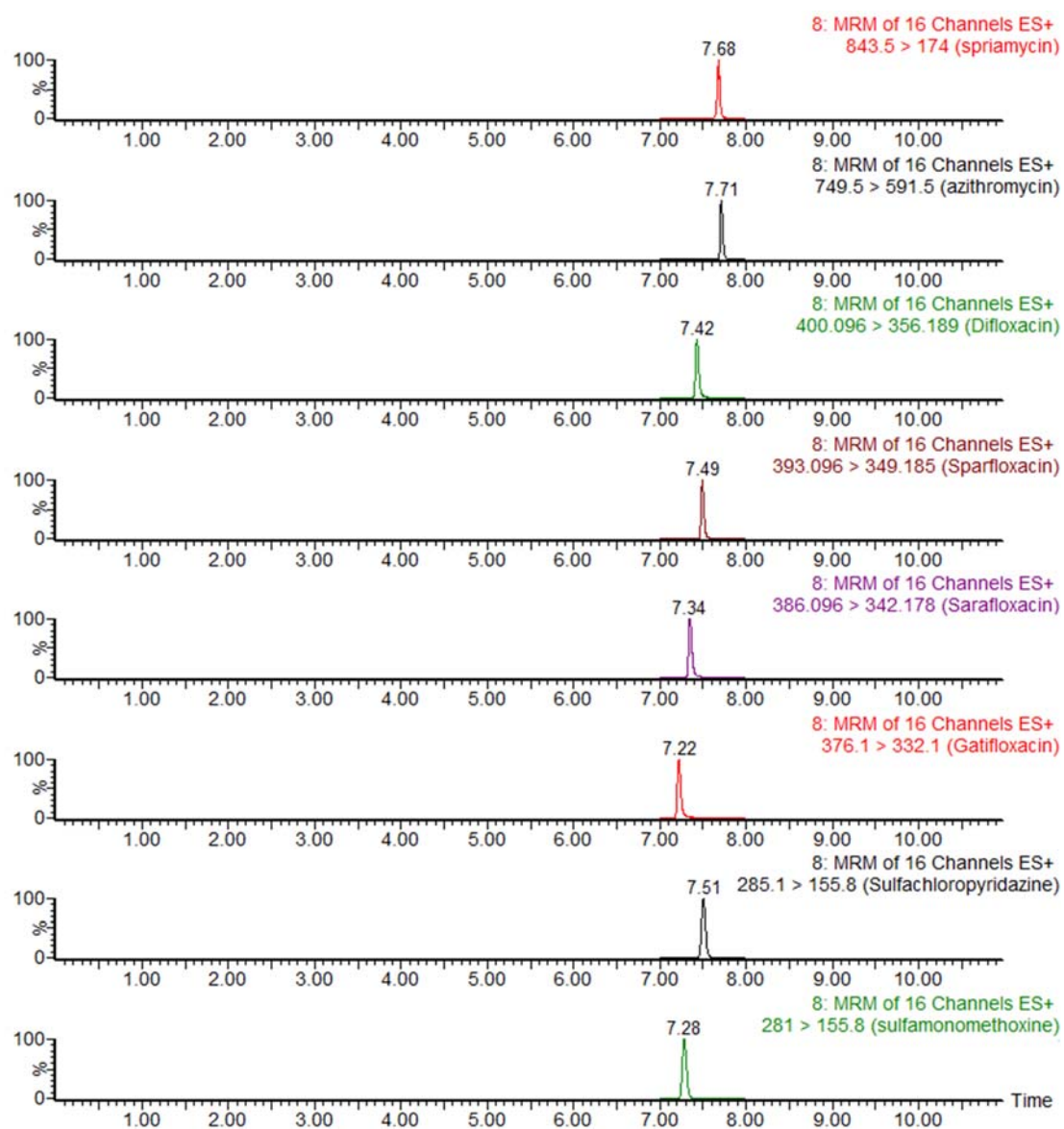

Fig s4. MRM chromatograms

Supplement: Supplementary file 4 [file Image_4.pdf]
